# Distinctive profiles of small RNA couple inverted repeat-induced post-transcriptional gene silencing with endogenous RNA silencing pathways in *Arabidopsis*

**DOI:** 10.1261/rna.046532.114

**Published:** 2014-12

**Authors:** Tadeusz Wroblewski, Marta Matvienko, Urszula Piskurewicz, Huaqin Xu, Belinda Martineau, Joan Wong, Manjula Govindarajulu, Alexander Kozik, Richard W. Michelmore

**Affiliations:** 1 The Genome Center, University of California, Davis, Davis, California 95616, USA; 2 Department of Plant Science, University of California, Davis, Davis, California 95616, USA; 3 Department of Molecular and Cellular Biology, University of California, Davis, Davis, California 95616, USA; 4 Department of Medical Microbiology and Immunology, University of California, Davis, Davis, California 95616, USA

**Keywords:** heterochromatic siRNA, transitivity, RNA decay, hairpin RNA, chalcone synthase, translation

## Abstract

The experimental induction of RNA silencing in plants often involves expression of transgenes encoding inverted repeat (IR) sequences to produce abundant dsRNAs that are processed into small RNAs (sRNAs). These sRNAs are key mediators of post-transcriptional gene silencing (PTGS) and determine its specificity. Despite its application in agriculture and broad utility in plant research, the mechanism of IR-PTGS is incompletely understood. We generated four sets of 60 *Arabidopsis* plants, each containing IR transgenes expressing different configurations of *uidA* and *CHALCONE SYNTHASE* (At-*CHS*) gene fragments. Levels of PTGS were found to depend on the orientation and position of the fragment in the IR construct. Deep sequencing and mapping of sRNAs to corresponding transgene-derived and endogenous transcripts identified distinctive patterns of differential sRNA accumulation that revealed similarities among sRNAs associated with IR-PTGS and endogenous sRNAs linked to uncapped mRNA decay. Detailed analyses of poly-A cleavage products from At-*CHS* mRNA confirmed this hypothesis. We also found unexpected associations between sRNA accumulation and the presence of predicted open reading frames in the trigger sequence. In addition, strong IR-PTGS affected the prevalence of endogenous sRNAs, which has implications for the use of PTGS for experimental or applied purposes.

## INTRODUCTION

Small RNAs (sRNA) in plants exhibit a range of size classes, predominantly from 20 to 24 nt, that are characteristic of their origin and the mechanism by which they are generated. Depending on their origin and structure, dsRNA triggers of RNAi are cleaved by one or more Dicer-like nucleases (DCLs) (Zamore et al. 2000; Bernstein et al. 2001); four DCLs are known in *Arabidopsis* (Henderson et al. 2006). DCL4 and DCL2 can process perfectly complementary dsRNA to produce 20- to 22-nt long sRNAs referred to as short-interfering RNAs (siRNAs) (Hamilton and Baulcombe 1999; Hamilton et al. 2002; Hannon 2002; Tang et al. 2003; Bouche et al. 2006). DCL1 cleaves partially paired dsRNA precursors to produce 21-nt long sRNAs known as microRNAs (miRNAs) (Denli and Hannon 2003; Bartel 2004) and DCL3 is involved in biogenesis of 24-nt long repeat-associated sRNAs known as heterochromatic siRNAs (hc-siRNAs) (Xie et al. 2005). When associated with one of the ARGONAUTE proteins (AGO) in an RNA-induced silencing complex (RISC), 20- to 21-nt long siRNAs and miRNAs direct sequence-specific cleavage of complementary target RNAs or interfere with their translation (Hammond et al. 2000; Hannon 2002; Brodersen et al. 2008). Twenty-one nucleotides long siRNAs may also participate in siRNA-dependent methylation of genomic loci (Pontier et al. 2012), a function traditionally attributed to the 24-nt long species and linked to chromatin modifications and transcriptional silencing (Hamilton et al. 2002; Zilberman et al. 2003; Liu et al. 2004; Law and Jacobsen 2010).

Endogenous dsRNA precursors of sRNA may be produced in eukaryotic cells through the transcription of self-complementary sequences and/or through the activity of RNA-dependent RNA polymerases (RDRs) that are capable of synthesizing complementary strands using preexisting single-stranded templates (Dalmay et al. 2000; Mourrain et al. 2000; Curaba and Chen 2008; Garcia et al. 2012). RDRs are also essential for the amplification of RNA silencing that frequently follows initial RISC-mediated cleavage of the target RNA and can result in the production of secondary sRNA. One such type of secondary sRNAs, known as transitive sRNAs, is produced from the sequences linked to the primary cleavage target during RNA silencing (Voinnet et al. 1998; Vaistij et al. 2002; Voinnet 2008). As with DCLs, particular RDRs are associated with the different pathways of sRNA biogenesis; for example, RDR2, along with DCL3, is critical for hc-siRNA production (Xie et al. 2004; Lu et al. 2006), and RDR6 generates perfectly complementary dsRNA substrates for DCL4 during biogenesis of transitive sRNA and *trans*-acting siRNA (ta-siRNA) (Peragine et al. 2004; Vazquez et al. 2004; Allen et al. 2005; Brodersen and Voinnet 2006). In *Arabidopsis*, amplification of RNA silencing requires not only RDR6 but also a helicase activity, which can be provided by enzymes like Silencing Defective 3 (SDE3) (Dalmay et al. 2001), a model for how enzymes may facilitate reuse of an original template and thereby accelerate amplification, as previously proposed (Garcia et al. 2012).

The various DCLs process dsRNAs that may occur in a cell as a consequence of viral infection or transgene expression (Chuang and Meyerowitz 2000; Llave et al. 2002). In these cases the trigger and the target are represented by the same sequence (trigger/target), but the sRNA products can also affect other RNAs present in the cell that share sufficient sequence identity with the trigger. Abundant dsRNA known as hairpin RNA (hpRNA) can be produced in plant cells through the expression of transgenes containing inverted repeats (IR) of the trigger sequence. This technique, referred to as IR post-transcriptional gene silencing (IR-PTGS), is commonly used to down-regulate endogenous mRNAs (Smith et al. 2000). Separation of inverted repeats by a functional intron sequence enhances the efficiency of PTGS; dsRNA produced from these transgenes is referred to as intron-spliced hairpin RNA (ihpRNA) (Smith et al. 2000). Although IR-PTGS has agricultural applications and is frequently used as a research tool, the mechanisms involved in ihpRNA processing are not well understood (Brodersen and Voinnet 2006; Brosnan and Voinnet 2011). Similarities between ihpRNA and the endogenous precursors of ta-siRNA (perfectly complementary dsRNA) as well as the abundant 21-nt long sRNAs associated with IR-PTGS, suggests the involvement of DCL4 (Gasciolli et al. 2005; Xie et al. 2005). In the absence of DCL4, DCL2 can process long, perfectly complementary dsRNAs that comprise endogenous DCL4 substrates to produce 22-nt long sRNAs (Gasciolli et al. 2005; Xie et al. 2005; Brodersen and Voinnet 2006). Forward genetic screens imply that short-distance signaling during IR-PTGS requires components of the endogenous 24-nt siRNA pathway (CLASSY1 [CLSY1], NUCLEAR RNA POLYMERASE D1a [NRPD1a], and RDR2) but not the components of the endogenous, RDR6-dependent, 21-nt siRNA pathway (Dunoyer et al. 2005; Smith et al. 2007; Brosnan and Voinnet 2011). Long-distance signaling involving vascular tissue requires components of both pathways (Brosnan et al. 2007; Dunoyer and Voinnet 2008; Jauvion et al. 2010).

Here we present a detailed analysis of sRNAs associated with IR-PTGS in *Arabidopsis*. We sequenced seven sRNA libraries produced from large numbers of transgenic plants expressing four different configurations of chimeric IR constructs designed to trigger RNA silencing of two different genes. Mapping of sRNA sequences to their corresponding transgene-derived and endogenous transcripts revealed distinctive patterns of sRNA accumulation. Analyses of these patterns linked endogenous pathways of RNA silencing with IR-PTGS and identified an unexpected correlation between the presence of ORFs in RNA sequences and sRNA accumulation. We also found that high levels of IR-PTGS affected endogenous levels of sRNAs, primarily by decreasing hc-siRNAs.

## RESULTS

### Configuration of the ihpRNA trigger sequence affects efficiency of IR-PTGS in *Arabidopsis*

**FIGURE 1.**
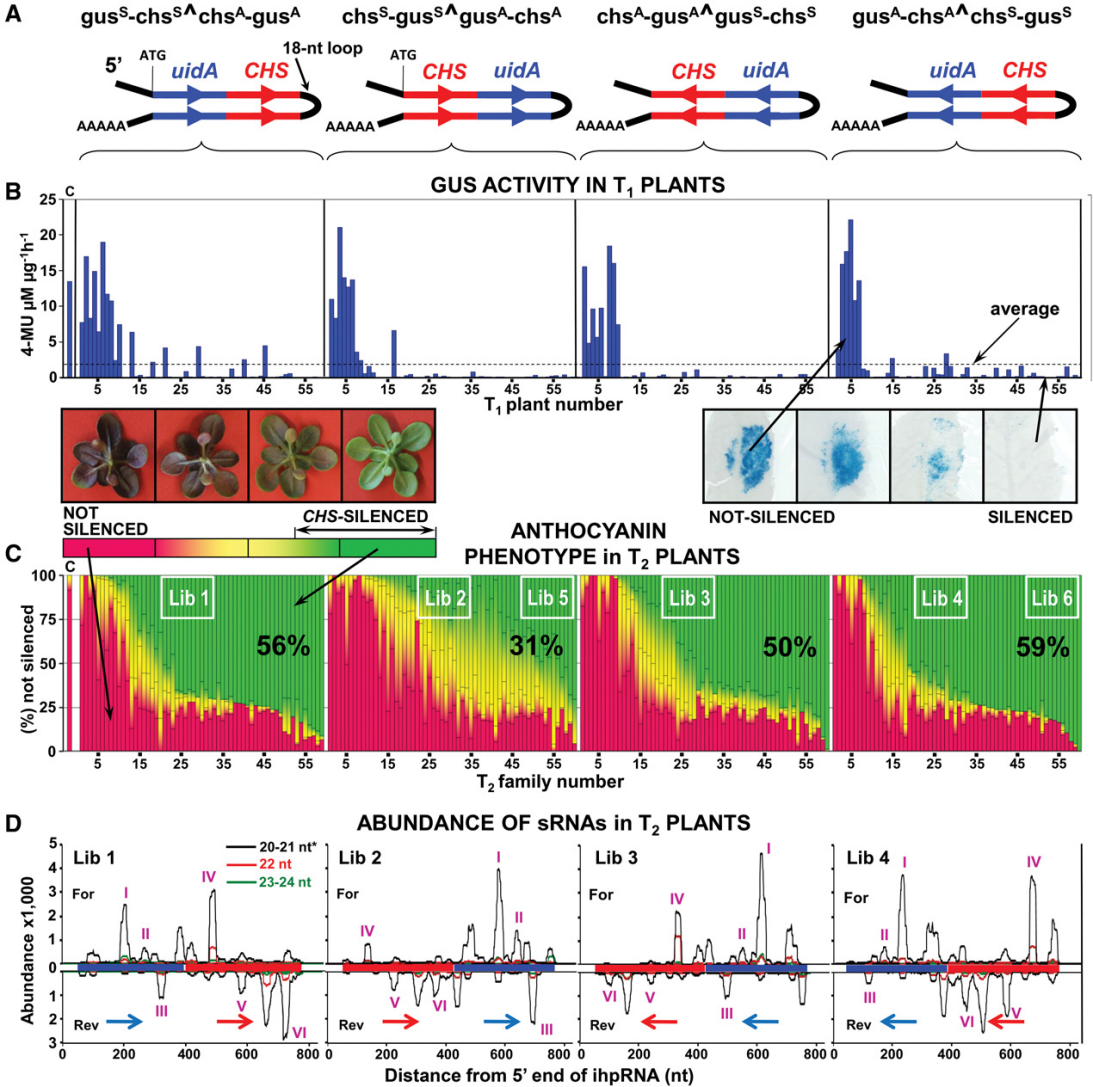
Phenotypes and sRNA profiles in transgenic *Arabidopsis* ecotype Ws-0 transformed with four constructs configured to produce different ihpRNAs. (*A*) Predicted ihpRNA structures produced by expression from the integrated T-DNA. (*B*) Expression of β-glucuronidase in the leaves of transgenic T_1_ plants measured after *Agrobacterium*-mediated transient expression of the *uidA* (*GUS*) gene. Each bar shows the average GUS activity per T_1_ plant. (*C*) Percentage of T_2_ plants derived from each T_1_ plant shown in (*B*) sorted into four classes depending on anthocyanin production: (red) not silenced, (red-yellow) weakly silenced, (yellow-green) moderately silenced, and (green) strongly silenced. The white rectangle indicates the plants used for construction of sRNA and RNA-Seq libraries. (c) control wild-type plants, *Arabidopsis thaliana* ecotype Ws-0. Each bar corresponds to 48 or more T_2_ individuals derived from each T_1_ plant shown in (*B*). (*D*) Abundance of transgene-derived sRNA sequences corresponding to the ihpRNA sequences. Roman numerals (purple) indicate peaks of sRNA accumulation compared between libraries. Abundance of sRNA in 1 million reads per library is shown. (*) 20-nt long sRNA accounted for up to 2% of the 20- to 21-nt ihpRNA-derived sRNA shown in the alignment.

To investigate the effects of ihpRNA configuration on the efficiency of IR-PTGS, we produced four sets of *Arabidopsis* transgenics (ecotype Ws-0), each transformed with one of four constructs designed to express ihpRNAs in different configurations. Each construct contained a 342-bp fragment of the bacterial *uidA* gene (Jefferson et al. 1987) fused to a 372-bp fragment of *Arabidopsis CHALCONE SYNTHASE* (At-*CHS*) in the sense (coding) and antisense orientations (Fig. 1A; Supplemental Fig. S1). Fifty-nine or 60 independent transgenic individuals were generated for each construct. All T_1_ plants were evaluated for their ability to silence *uidA* expression based on the level of transient ectopic expression of β-glucuronidase (GUS) (Fig. 1B). The T_2_ progenies of these plants were assayed for silencing of At-*CHS*, based on their ability to produce anthocyanin under stressful conditions (Fig. 1C).

Silencing of GUS in T_1_ plants was strongly correlated with silencing of At-*CHS* in the corresponding T_2_ progenies (*r* = 0.83; *P* ≥ 0.01); however, the efficiency of silencing each gene was influenced by the configuration of the trigger sequence in the ihpRNA construct. Silencing of GUS in T_1_ plants was less effective (*P* ≤ 0.1) when the *uidA* trigger sequence was close to the 5′ end of the ihpRNA and in the sense (coding) orientation (gus^S^-chs^S^^chs^A^-gus^A^; ^S^ and ^A^ = sense and antisense orientation of the fragment, respectively, ^ = the position of the intron) (Supplemental Fig. S1) as compared with plants transformed with the other three constructs. Similarly, silencing of At-*CHS* was significantly less effective (*P* ≤ 0.05) when the corresponding sequence was located closer to the 5′ end of the ihpRNA (chs^S^-gus^S^^gus^A^-chs^A^) in the sense (coding) orientation, as compared with the other three constructs. Thus, both the presence of the ORF and the position of the sequence in the ihpRNA affected efficiency of IR-PTGS.

### Configuration of ihpRNA trigger affects sRNA accumulation

To determine whether the observed differences in silencing were associated with differences in sRNA accumulation, we performed deep sequencing of seven sRNA libraries. For each construct, sRNAs were extracted from a pool of 120 T_2_ plants that exhibited intermediate (Lib 1–4) and strong levels of silencing (Lib 5 and Lib 6) (Fig. 1C). The sRNAs extracted from wild-type, nontransgenic plants were used for the reference library (Lib 0). Approximately 6 million sRNA sequences were obtained from each library using Illumina sequencing. The ihpRNA-derived sRNAs represented between 5.7% and 9.6% of the total sRNA in each library; most (80.0%–87.9%) were 20- to 21-mers, consistent with previous reports of involvement of this size class of sRNA in dsRNA-mediated silencing (Hamilton et al. 2002; Llave et al. 2002; Dunoyer et al. 2005). Two other abundant classes of transgene-derived sRNAs were 22-mers accounting for 7.9%–13.6% and 23- to 24-mers accounting for 1.8%–5.2% of all sRNAs in each library.

**FIGURE 2.**
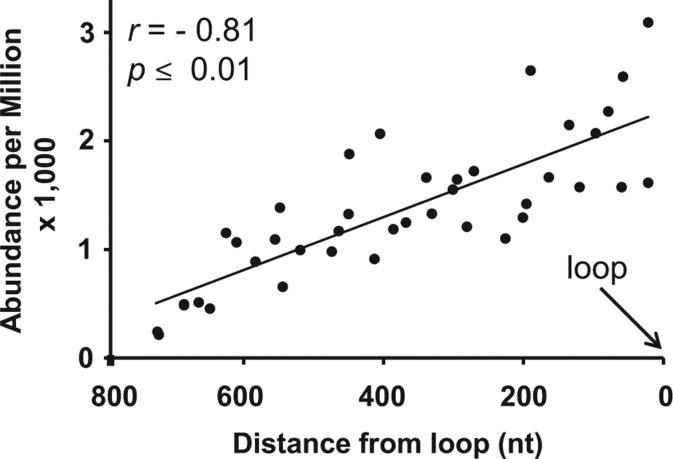
Correlation between the abundance of transcripts in ten major peaks of sRNA production and the position of these peaks in the ihpRNA. The five largest peaks corresponding to the At-*CHS* and *uidA* (*GUS*) fragments were selected and the abundance was calculated for all four sRNA libraries (Libs 1–4). (*r*) correlation coefficient.

BLAST analysis and mapping of the sRNAs to the corresponding ihpRNA sequences revealed peaks of sRNA accumulation in both *uidA* and At-*CHS* sequences, rather than an even or random distribution across the length of the trigger sequences (Fig. 1D). The positions of these peaks with respect to each trigger sequence were the same for all four ihpRNAs, indicating some form of dependence on the sequence (see below) rather than the configuration of the ihpRNA construct (Fig. 1D). However, the abundance of sRNAs associated with each peak varied substantially depending on the position and the orientation of the trigger sequence within the construct (Fig. 1A,D). Significantly fewer sRNAs were produced when the fragment of At-*CHS* or *uidA* was adjacent to the 5′ end of the ihpRNA in the sense (coding) orientation, compared with the antisense orientation (*P* ≤ 0.01). When the trigger fragment was adjacent to the intron/loop, sRNAs were produced at higher levels regardless of sequence orientation. Accordingly, the abundance of sRNAs associated with each peak was inversely correlated (*r* = −0.81; *P* ≤ 0.01) with the sequence's distance from the loop/intron (Fig. 2). These construct-dependent differences in sRNA accumulation paralleled the phenotypic differences in GUS and *At*-CHS silencing; higher sRNA accumulation was correlated with stronger silencing. From this experiment we conclude that levels of sRNA production and IR-PTGS efficiency are highest when the trigger sequence is closer to the loop in ihpRNA. In addition, the presence of an ORF in the first (5′ most) arm of IR decreases sRNA production and efficiency of IR-PTGS.

### ihpRNA-derived sRNAs and sRNAs linked to uncapped mRNA decay are similar, and their accumulation is associated with the presence of predicted ORFs in the trigger sequence

**FIGURE 3.**
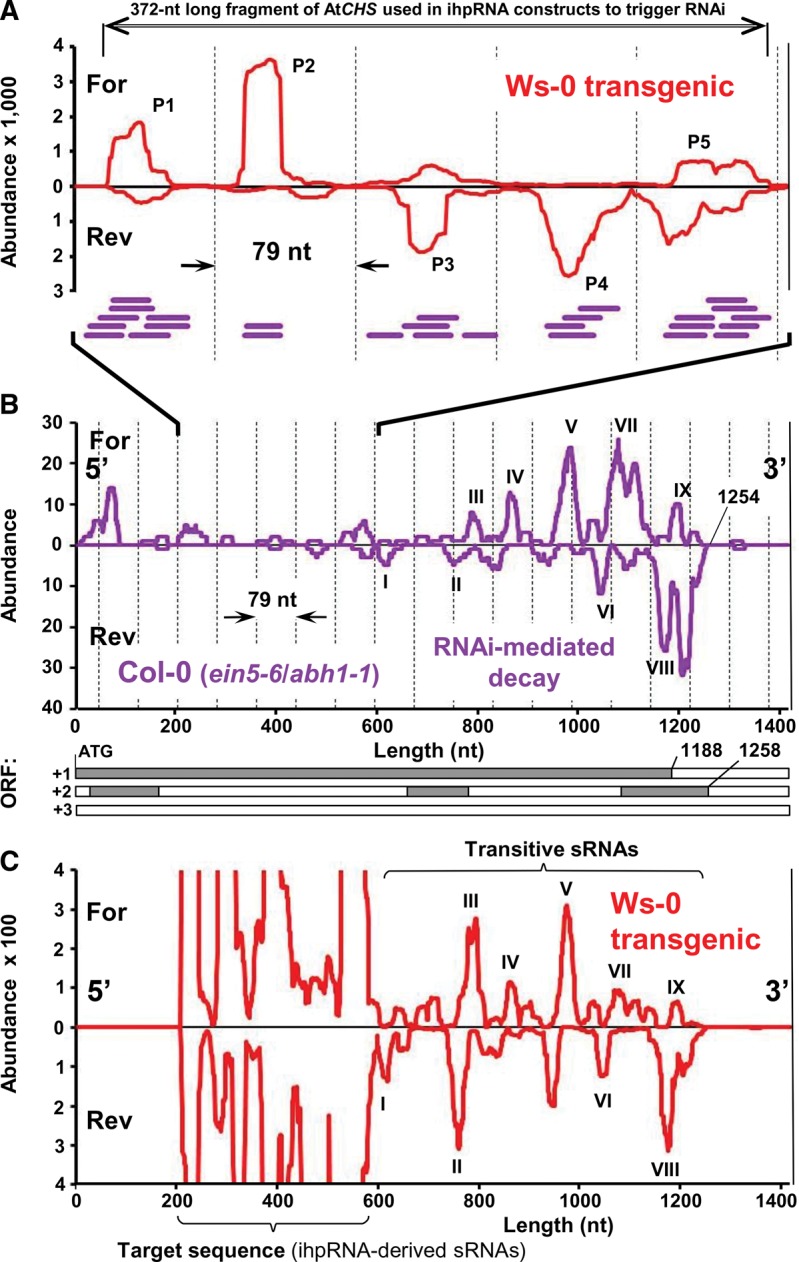
Analysis of endogenous, transgene-derived and transitive sRNAs corresponding to At-CHS* in *Arabidopsis*. (*A*) Similarity in the distribution of ihpRNA-derived sRNAs (red) in transgenic Ws-0 plants and sRNA associated with uncapped mRNA decay in the *ein5-6*/*abh1-1* mutant in Col-0 ecotype background (purple bars). Sequences of sRNA were mapped to the fragment of At-CHS* sequence used in the ihpRNA construct. (*B*) Abundance of nonredundant sRNA sequences corresponding to the full-length At-CHS* identified in Col-0 (*ein5-6*/*abh1-1* mutant) (Gregory et al. 2009). sRNAs match the 3′ fragment of the At-CHS* mRNA that corresponds to the primary and secondary ORFs in frame 1 and 2, respectively. Roman numerals indicate peaks of sRNA accumulation for comparison to transitive sRNAs shown in (*C*). (*C*) Representation of sRNA transcripts corresponding to the full-length At-CHS* in transgenic Ws-0 plants showing numerous transitive sRNAs matching the region downstream from (3′) but not upstream of (5′) the RNAi target sequence. Abundance corresponds to representation per nucleotide per million reads. Since the abundance of At*CHS* mRNA-derived sRNA did not exceed 300 per million per nt and the abundance of reads corresponding to the trigger/target fragment approached 3500 per million per nucleotide, we assumed that sRNAs corresponding to the trigger/target fragment originated from ihpRNA processing. *mRNA sequences of At-*CHS* in ecotypes Col-0 and Ws-0 are identical.

To investigate whether ihpRNA-derived sRNAs in our experiments were similar to those produced endogenously, we searched the control library (Lib 0) generated from wild-type ecotype Ws-0 plants for At-*CHS*-derived sRNAs. Among over 6 million reads in this library we found only one 21-mer corresponding to a region of the At-*CHS* mRNA upstream of the trigger fragment used in our ihpRNA constructs. In contrast, we retrieved abundant 20- to 21-nt long At-*CHS*-derived sRNAs from the publicly available sRNA database for *Arabidopsis* ecotype Col-0 (http://mpss.udel.edu/at_sbs/). Interestingly, these sRNAs in ecotype Col-0 were very similar to the highly abundant sRNA species corresponding to the At-*CHS* trigger fragment in our transgenic Ws-0 plants; i.e., both sets of sRNAs mapped to the same peaks in the At-*CHS* sequence that occurred with an approximate periodicity of 79 nt (Fig. 3A,B).

Over 65% of the At-*CHS*-derived sRNAs from Col-0 in the database originated from a library made from the *ein56*/*abh11* mutant, which is defective in mRNA capping and deficient in the exoribonuclease 4 (XRN4) responsible for 5′→3′ degradation of uncapped mRNA (Gregory et al. 2009). The *ein56*/*abh11* mutations caused elevated sRNA production and mRNA degradation for 133 distinct genes, one of which is At-*CHS* (Gregory et al. 2009). Interestingly, sRNAs from the *ein56/abh11* mutant precisely (within a few nucleotides) matched the fragment of the At-*CHS* mRNA corresponding to reading frame 1 and to a secondary predicted ORF in reading frame 2 that extends past the first ORF (Fig. 3B). To investigate this phenomenon further, we analyzed all of the 133 genes affected by RNAi-mediated decay in this *ein56/abh11* mutant (Gregory et al. 2009). For 90 of these genes, accumulation of sRNA was primarily associated with predicted primary or secondary ORF sequences (Supplemental Fig. S2). For the remaining 43 genes, the representation of corresponding sRNAs was too low for analysis. Therefore, ihpRNA-derived sRNAs in our transgenic plants are similar to the endogenous sRNAs associated with mRNA decay, and the accumulation of the latter appears to be influenced by the presence of ORFs in the target RNA.

### Patterns of 3′ transitive sRNA production match patterns of sRNA accumulation associated with uncapped mRNA decay

Induction of RNA silencing has been reported to trigger production of secondary, transitive sRNAs from the regions flanking the target sequence (Voinnet et al. 1998; Vaistij et al. 2002; Moissiard et al. 2007; Voinnet 2008). We searched our sRNA libraries for secondary sRNAs corresponding to regions of the full-length At-*CHS* mRNA outside of the trigger/target sequence used in our ihpRNA constructs. In the libraries made from transgenic plants (Libs 1–4), we found numerous sRNAs matching the region downstream, but not upstream of the trigger/target sequence (Fig. 3C). Interestingly, the pattern of these transitive sRNAs corresponded exactly to the pattern of sRNAs derived from native At-*CHS* in the ecotype Col-0 *ein5-6/abh1-1* mutant described above (Fig. 3B,C). Therefore we concluded that the mechanism of sRNA biogenesis may be the same in both experimental systems: (1) RNAi-mediated degradation of uncapped mRNA in the absence of a 5′→3′ exoribonuclease in the *ein5*/*abh1-1* mutant and (2) 3′ transitivity in our Ws-0 transgenics.

To validate this hypothesis we analyzed At-*CHS*-derived transcripts in transgenic and wild-type control plants before and after treatment with 5′-phosphate-dependent exonuclease (5′PDE; catalog # TER51020; www.epibio.com) to degrade uncapped RNA. We constructed 14 RNA-Seq libraries using poly-A RNA purified from five total-RNA preps used to make sRNA libraries (see Materials and Methods). Seven libraries designated ESeq 0–6 and seven designated RSeq 0–6 were constructed from 5′PDE-treated and untreated poly-A RNA, respectively. Libraries ESeq 0–6 and RSeq 0–6 were made from the same total RNA preps as the sRNA libraries 0–6, respectively (Fig. 1C). Nine million reads from each library were aligned to *Arabidopsis* rRNA-5S-encoding sequences, 13 reference sequences used previously for transcript normalization (Czechowski et al. 2005), and the At-*CHS* mRNA sequence. Read alignment to the rRNA-5S-encoding sequences revealed a 52% reduction in mean abundance (reads per kilo base pair per million) from ESeq versus RSeq libraries, consistent with 5′PDE-mediated degradation of residual rRNA in the poly-A RNA samples used for ESeq library construction (Supplemental Table S2). Alignment to 13RS mRNA reference sequences used for normalization showed an average of 16% increase in abundance (*P* ≤ 0.05) from ESeq versus RSeq libraries (Supplemental Table S2), consistent with 5′PDE-mediated removal of uncapped RNAs during ESeq library construction and a corresponding increase in the relative abundance of species that are insensitive to 5′PDE-mediated degradation. Finally, read alignment to the At-*CHS* mRNA showed that abundance increased by 19% in ESeq 0 compared with RSeq 0, indicating that At-*CHS* mRNA in wild-type plants contained the protective 5′ phosphate cap. The insensitivity of At-*CHS* mRNA to 5′PDE-mediated degradation in vitro was consistent with its insensitivity to RNAi-mediated degradation in vivo, as indicated by lack of its corresponding sRNA in Lib 0.

**FIGURE 4.**
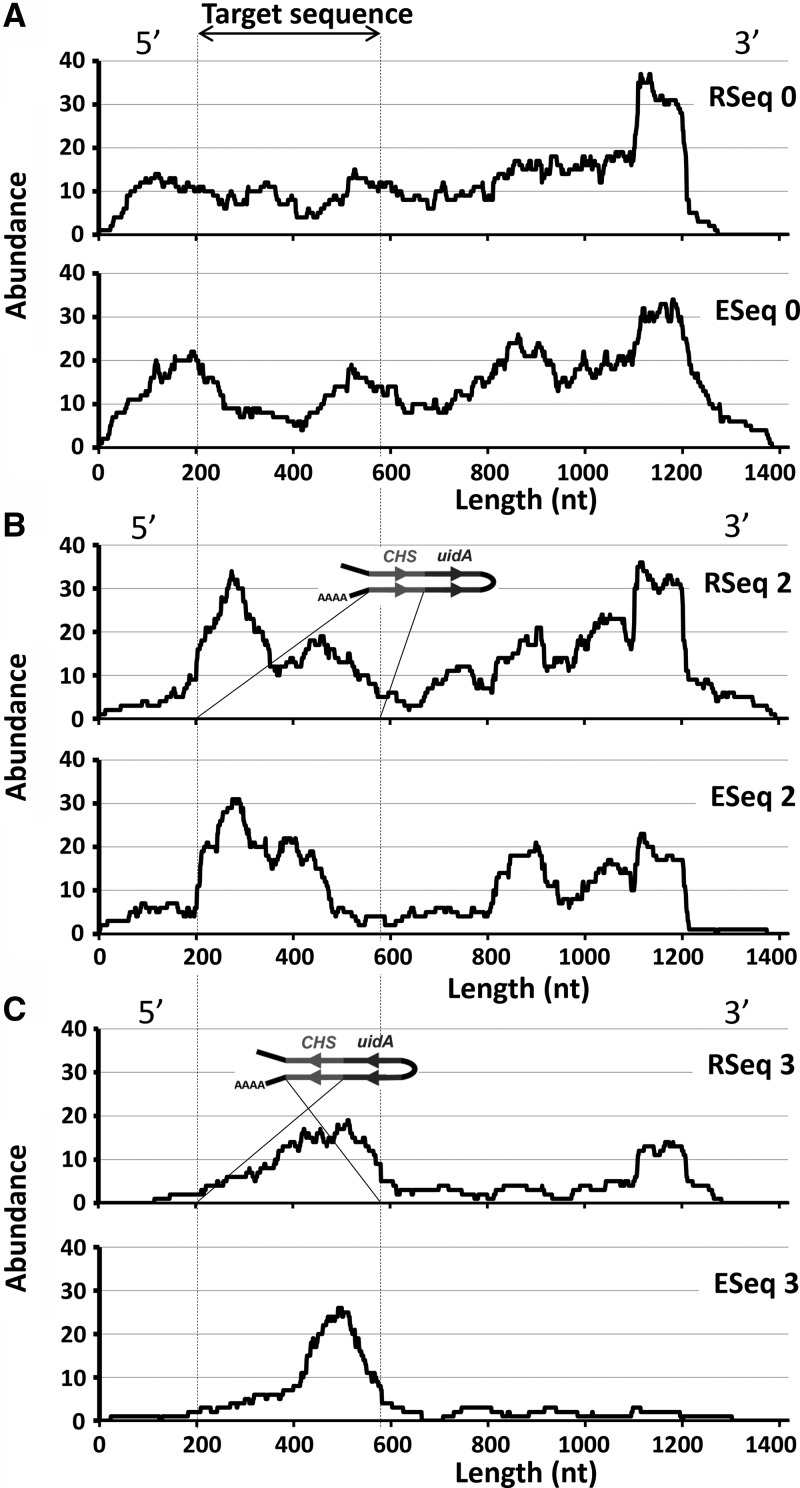
Alignment of reads from RSeq and ESeq libraries to the sequence of At*CHS* mRNA. (*A*) In wild-type plants the reads were distributed across the entire length of At*CHS* mRNA sequence; treatment with 5′PDE (ESeq 0) resulted in a slight increase in the coverage. (*B*) In RSeq 2, made from the plants transformed with chs^S^-gus^S^^gus^A^-chs^A^ and least efficient in silencing of At*CHS* mRNA, the read coverage upstream (5′) of the target sequence is clearly reduced, but the coverage downstream (3′) is comparable to that in wild-type plants. The reads corresponding to the 3′ fragment originated from RNA sensitive to 5′PDE treatment, as indicated by reduced coverage in ESeq 2. (*C*) Even greater reduction in At*CHS* mRNA-derived reads was observed among plants transformed with chs^A^-gus^A^^gus^S^-chs^S^. In both (*B*) and (*C*) the transgene-derived reads are more abundant toward the predicted poly-A tail within the ihpRNA structure. Coverage indicates number of corresponding reads per nucleotide out of 9 million total reads per library.

Further evidence for 5′-capped full-length At-*CHS* mRNA and uncapped products of its cleavage comes from the distribution of RNA-Seq read alignments to the At-*CHS* sequence. Reads from the wild-type plant libraries (ESeq 0 and RSeq 0) aligned along the entire length of the At-*CHS* mRNA sequence (Fig. 4A), in contrast to reads from transgenic plant libraries (RSeq 1–6 and ESeq 1–6), for which several trends were observed. First, the increased relative abundance of reads matching the trigger/target fragment in the IR constructs indicates that the ihpRNA transcripts were polyadenylated and hence copurified along with native mRNAs and that they were incompletely processed to produce sRNAs (Fig. 4B,C). As is consistent with the progressive processing of ihpRNA starting from the loop, the distribution of these aligned reads showed clear bias for areas adjacent to the poly-A tail (Fig. 4B,C). Second, the reduction or lack of reads aligning upstream of the trigger/target sequence (Fig. 4B) in contrast to their relative abundance downstream indicates the cleavage of At*CHS* mRNA within this region. This bias can be attributed to reduced representation of cleaved 5′ ends in poly-A-selected mRNA during library construction. Third, a relative decrease in reads corresponding to the 3′ At*CHS* mRNA fragment in ESeq versus RSeq libraries (Fig. 4B,C) indicates a deficiency in 5′ phosphate groups that results in sensitivity to 5′PDE-mediated degradation; this contrasts with an expected increase in coverage as described above for capped full-length At-*CHS* mRNAs (RSeq 0 versus ESeq 0) (Fig. 4A) and 13RS in all ESeq libraries.

We concluded that in the transgenic plants, ihpRNA-derived sRNAs mediate cleavage of the corresponding At-*CHS* mRNA, generating 3′ uncapped fragments that are directed to the RNAi-mediated decay pathway (Gazzani et al. 2004; Souret et al. 2004; Gregory et al. 2009). In addition, the similarity of sRNA profiles we observed indicates that 3′ transitivity and silencing-mediated RNA decay involve the same processes. In contrast, the 5′ fragment of the cleavage reaction is likely to be degraded by the exosome (Parker and Song 2004); accordingly, we did not find any evidence for primer-dependent 5′ spreading of sRNA production that would be indicative of 5′ transitivity (Fig. 3C; Sijen et al. 2001; Voinnet 2008).

### Position of peaks of sRNA accumulation may be determined by rare codons

Our data indicated that sRNA production was primarily associated with the presence of ORFs. Therefore, we explored characteristics of the trigger sequence that might correlate with the observed peaks of sRNA accumulation. Previous reports (De Paoli et al. 2009; Kasai et al. 2013) suggested that secondary structures in *CHALCONE SYNTHASE A* (*CHS-A*) mRNA may trigger production of both endogenous and transgene-derived sRNAs during sense-mediated PTGS (S-PTGS) in petunia (Napoli et al. 1990). Consistent with this proposal, one of the five major peaks (P2) (Fig. 3A) in our At-*CHS* trigger sequence corresponded to an extended area of predicted self-complementarity in At-*CHS* mRNA that additionally resembles pre-microRNA (Supplemental Fig. S3). However, because our analyses showed that the biogenesis of sRNAs was associated with the presence of ORFs, we also examined whether the presence of rarely utilized codons coincided with sRNA accumulation. We adapted a previously published algorithm (Clarke and Clark 2008) to calculate the relative codon frequency for each amino acid in *Arabidopsis* based on codon usage (http://www.kazusa.or.jp/codon/) and assigned a rarity value to each codon.

**FIGURE 5.**
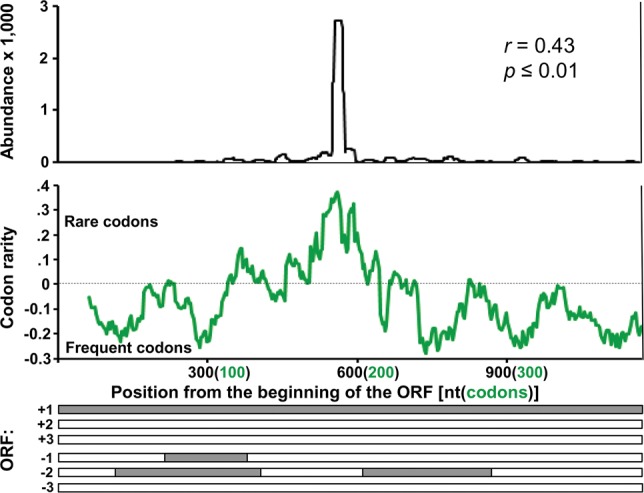
Distribution of sRNA associated with S-PTGS in petunia. Abundance of sRNAs mapped to the petunia *CHS-A* sequence (De Paoli et al. 2009) (*top* panel) and the frequency of rare codons in the sequence (*middle* panel) showing that the peak of sRNA production is correlated with a high abundance of rare codons and is independent of predicted ORFs (*bottom* panel). (*r*) correlation coefficient.

For the At-*CHS* fragment used in our ihpRNA constructs we did not find a correlation between sRNA accumulation and codon frequency; however, the regions in the ihpRNA *uidA* fragment corresponding to peaks of sRNA were weakly but significantly (*P* ≤ 0.01) correlated with elevated frequencies of rare codons (Supplemental Fig. S4). We performed similar analyses on the *CHS-A* gene from petunia using relative codon frequencies calculated for this species. Most of the *CHS-A* ORF comprises frequently used codons in petunia; however, a small central region contains a substantially elevated abundance of rare codons (Fig. 5). This region coincides precisely with the most abundant transgene-derived sRNAs in petals of cosuppressed petunia plants (Fig. 5). Because the full-length *CHS-A* mRNA had no strong detectable sequence complementarities near the major peak for sRNA accumulation (data not shown), the peak's position appears more consistent with the elevated frequency of rare codons than with complementarity in the predicted mRNA secondary structure as suggested previously (De Paoli et al. 2009; Kasai et al. 2013).

### IR-PTGS affects the prevalence of endogenously produced sRNAs

**FIGURE 6.**
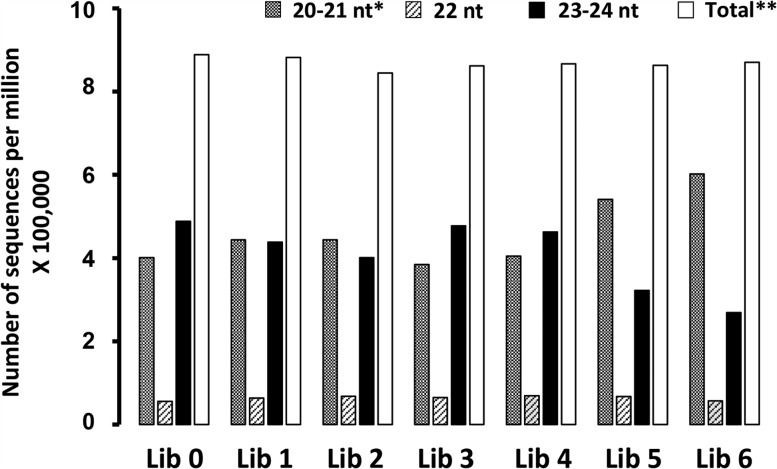
Distribution of different size classes of endogenous sRNA in the control library (Lib 0), four libraries made from moderately silenced plants (Libs 1–4), and two libraries made from strongly silenced plants (Libs 5 and 6). A clear increase in 20- to 21-nt long species and a decrease in 23- to 24-nt long species occur in strongly silenced plants. (*) All 20- to 24-nt long sequences matching the sequence of five *Arabidopsis* chromosomes. (**) 20-nt long sRNA accounted for roughly one-third to half of the 20- to 21-nt size class.

Processing of highly abundant, transgene-derived ihpRNAs engages the molecular components normally involved in biogenesis of endogenous sRNA. To evaluate the effect of IR-PTGS on endogenous sRNA accumulation, we analyzed the size distribution of sRNAs in four libraries made from moderately silenced plants (Libs 1–4), two libraries made from strongly silenced plants (Libs 5 and 6) (Fig. 1), and the reference library made from wild-type plants (Lib 0). In Lib 0, the number of 20- to 21-nt long species approached 401,000 sequences per million (tspm) compared with 488 tspm of the 23- to 24-nt long species (Fig. 6). Among moderately silenced plants (Libs 1–4), the average abundance of 20- to 21-nt long sRNA species was still lower than the 23- to 24-nt long species at 419 and 445 tspm, respectively. However, in both libraries obtained from strongly silenced plants (Libs 5 and 6), the number of 20- to 21-nt long reads increased to 541 and 602 tspm (1.4- and 1.5-fold increases, respectively) and the number of 23- to 24-nt long reads decreased to 322 and 269 tspm (1.5- and 1.8-fold decreases, respectively) (Fig. 6). The difference could not be attributed to the additional 20- to 21-nt long transgene-derived sRNAs because their abundance did not exceed 10% of the total number of sRNAs in any library analyzed.

**FIGURE 7.**
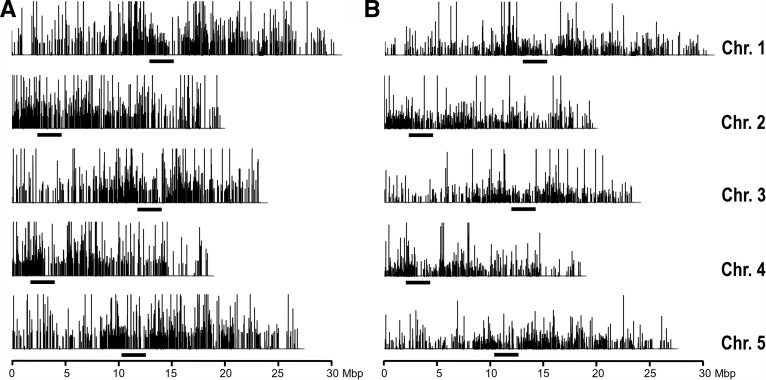
Chromosomal distribution of 23- to 24-nt long sRNAs in *Arabidopsis* plants. (*A*) Wild-type control plants (Lib 0) and (*B*) strongly silenced plants (Lib 6). Bars indicate the approximate positions of the centromeres.

To identify particular sRNA species responsible for observed differences, we mapped the sRNAs from three libraries (Libs 0, 5, and 6) to the five *Arabidopsis* chromosomes. Next, assuming that sRNA causing the size shift may represent only a fraction of the size class, we searched for loci for which the levels of corresponding sRNA differed by >1.5-fold between strongly silenced plants (Libs 5 and 6) and the control plants (Lib 7). Loci were defined as fragments of the reference sequence in which at least 10 sequential nucleotides were represented by at least 10 sequences per million in each of the three libraries analyzed. Differences were considered significant when they were detected in both the comparisons of Lib 5–Lib 0 and Lib 6–Lib 0. We identified 141 loci for which the corresponding 20- to 21-nt long sRNAs were over-represented in strongly silenced plants (Supplemental Fig. S5). These comprised 22 loci encoding known miRNAs, including MIR156b, and five loci encoding tasiRNAs, one of which was TAS2 (Peragine et al. 2004; Vazquez et al. 2004; Allen et al. 2005; Jones-Rhoades et al. 2006). We also identified 43 loci in which the corresponding 20- to 21-nt long sRNAs were under-represented in strongly silenced plants (Supplemental Fig. S6); among these were four loci that encode transcripts known to be the targets of TAS2 and MIR156b. This provides biological support for the observed increases in the abundance of these two regulatory sRNAs (Supplemental Fig. S5). Among the loci responsible for the production of 23- to 24-nt long species, we found 36 for which the number of corresponding sRNAs was increased. Most importantly, however, 3086 loci were found for which corresponding sRNAs were decreased in strongly silenced plants. Mapping of these sRNAs to the sequences of the five *Arabidopsis* chromosomes (Fig. 7) showed their prevalence in pericentromeric regions, characteristic of hc-siRNA (Lu et al. 2006). Abundance of sRNAs corresponding to 2131 of these 3086 loci decreased by more than twofold, which is more than the overall 1.5- to 1.8-fold decrease in 23- to 24-nt long sRNA species we observed in strongly silenced plants. Because hc-siRNAs contribute substantially to the overall prevalence of the 23- to 24-nt long sRNA species in *Arabidopsis*, we concluded that strong IR-PTGS affects the prevalence of endogenous sRNAs, primarily by decreasing accumulation of 23- to 24-nt long hc-siRNAs.

## DISCUSSION

IR-PTGS is a commonly used approach to experimentally down-regulate gene expression in plants. Contemporary guidelines for designing constructs to maximize the efficiency of silencing have often been inferred from isolated successful cases and/or limited numbers of transgenic events. Here we provide extensive evaluation of four different constructs specifically designed to investigate the efficacy of RNAi induction based on 239 independent T_1_ transgenic plants, several thousand transgenic progenies, and detailed analyses of the different RNA species associated with silencing. We conclude that to maximize the efficiency of IR-PTGS, the first (5′-most) arm of the IR should contain the trigger sequences in an antisense orientation. The sequences closer to the intron separating the arms of the IR produce more sRNAs and therefore trigger PTGS more efficiently (Fig. 1).

In our experiments, the majority of the transgene-derived sRNAs were produced from the 500- to 600-nt long region adjacent to the loop of the ihpRNA, and the relationship of sRNA abundance to distance from the loop (Fig. 2) implies that the use of trigger sequences totaling >800 nt is undesirable. Accordingly, if multiple genes are to be silenced using a single chimeric ihpRNA, the length of each sequence should be adjusted proportionally depending on its position in the ihpRNA. The enhanced sRNA production from fragments of ihpRNA adjacent to the predicted loop (Fig. 2) may reflect the dynamics of dsRNA assembly. The two complementary RNA strands may achieve physical proximity more quickly at the splicing junction, resulting in formation of the dsRNA that subsequently triggers production of abundant sRNAs.

In earlier reports we implemented silencing of transiently expressed GUS as a reporter for silencing used in multiple generations of transgenic lettuce plants by incorporating a fragment of the *uidA* gene into the chimeric ihpRNA construct (Wroblewski et al. 2007). Using information presented in this manuscript, the system was further optimized by moving the fragment of the *uidA* gene to the 5′ end of the ihpRNA and reducing its length from 342 to 300 bp. This made silencing of GUS less effective but also more indicative and reliable as a reporter for simultaneous IR-PTGS of the targeted gene. This improved configuration of the ihpRNA construct enabled us to successfully use reverse genetics to dissect complex disease resistance loci and demonstrate host-induced gene silencing as a strategy for resistance to biotrophic pathogens in lettuce (M Christopoulou and M Govindarajulu, unpubl.).

Several previously reported observations place the RNAi process in the nuclear compartment of the plant cell. DCL4, which is primarily involved in the processing of perfectly complementary dsRNAs such as those comprising ihpRNA, resides exclusively in the nucleus (Kumakura et al. 2009; Hoffer et al. 2011; Jouannet et al. 2012). In addition, intron sequences alone have been sufficient to trigger IR-PTGS, suggesting that not only ihpRNA processing but the entire process of IR-PTGS can be carried out in the nucleus as well (Hoffer et al. 2011). Consistent with nuclear positioning of the RNAi machinery, extensive IR-PTGS in our transgenic plants considerably altered levels of endogenous sRNA produced in the nucleus. Nucleoli are considered to be the processing centers for both DCL3-dependent hc-siRNAs and DCL1-dependent miRNAs (Pontes and Pikaard 2008), levels of which showed significant changes in our strongly silenced plants (Fig. 7; Supplemental Figs S5, S6).

The high levels of dsRNA produced during IR-PTGS have been suggested to promote the activities of DCLs that would normally act in other RNA silencing pathways (Brodersen and Voinnet 2006). DCL4 and DCL2, for example, are both capable of processing long, perfectly complementary dsRNA (Brodersen and Voinnet 2006). DCLs are associated with the production of particular sRNA size classes (Brodersen and Voinnet 2006) and DCL3 is normally involved in the biogenesis of hc-siRNAs that are 24-nt long. Therefore, DCL3 is unlikely to have been recruited to process ihpRNA in our experiments since ihpRNA-derived sRNAs are primarily 20- to 21-nt long. Instead, the increase of more than two dozen specific miRNAs and ta-siRNA species (Supplemental Fig. S5) and the profound decrease of 23- to 24-nt long hc-siRNA species (Fig. 6) in our strongly silenced *Arabidopsis* plants resemble changes in endogenous sRNA production observed in the absence of RDR2 (Lu et al. 2006). This would imply, at least in the strongly silenced plants, the involvement of RDR2 or another molecular component of the RDR2-dependent sRNA pathway in ihpRNA processing. Interestingly, RDR2, as well as other components of the hc-siRNA pathway such as CLSY1 and NRPD1a, has previously been linked to short-distance signaling during IR-PTGS (Dunoyer et al. 2005, 2007; Smith et al. 2007; Brosnan and Voinnet 2011).

Another interesting observation in our experiments is that sequence translatability has a negative quantitative effect on sRNA production from ihpRNA, which is contradictory to the notion of IR-PTGS occurring in the nucleus since canonical translation takes place in the cytoplasm. The overall decrease in sRNA accumulation associated with the presence of ORFs, particularly from the 5′ fragment of ihpRNA (Fig. 1), suggests some level of translation and competition between the translation and RNAi machineries. A competition for single-stranded mRNA templates during RNA-mediated uncapped mRNA decay has been previously proposed (Gazzani et al. 2004; Gregory et al. 2009) and it was attributed to the limited access of RDR to the mRNA being translated and its inability to synthesize the complementary strand. In theory, because DCL-mediated processing of ihpRNA should not require synthesis of the complementary strand, the initiation of translation and association of ihpRNA with ribosomes may simply prevent its folding to form the double-stranded template for DCL. However, the observations discussed below suggest that processing of ihpRNA to produce sRNA may be more complex than simple DCL4- or DCL2-mediated cleavage of dsRNA.

Mapping of ihpRNA-derived sRNAs in our transgenic Ws-0 plants to their corresponding sequences revealed clearly delineated peaks (Fig. 3). The positions of these peaks cannot be explained by sequential DCL4-mediated cleavage because they were not dependent upon ihpRNA configuration. Although both fragments were linked together in one ihpRNA molecule, the positions of the peaks corresponding to the At-*CHS* fragment were consistent and completely independent of the positions of the peaks corresponding to the *uidA* fragment. In addition, the five major peaks corresponding to the At-*CHS* gene fragment were distributed in approximate 79-nt intervals (Fig. 3A,B), which does not fit with the 21-nt phasing observed after sequential DCL4-mediated cleavage (Napoli et al. 1990; Howell et al. 2007; De Paoli et al. 2009; Kasai et al. 2013). In fact, the positions of the peaks corresponding to the At-*CHS* fragment used to generate ihpRNA were the same as those of the peaks produced by sRNAs associated with uncapped RNA decay of At-*CHS* mRNA in the *ein5*/*abh1-1* mutant (Gregory et al. 2009). This suggests that the determinant(s) of the positions of sRNA peaks in uncapped mRNA decay and ihpRNA processing are likely the same.

To investigate the determinants of the positions of sRNA peaks observed during IR-PTGS, RNAi-mediated ssRNA decay and S-PTGS, we evaluated several factors. We investigated the 5′ terminal nucleotides that may affect the ability of sRNA to bind AGO (Mi et al. 2008) but we were not able to find a clear preference for any particular nucleotide (data not shown). This was consistent with peaks being composed of overlapping and diverse sRNA species. Examination of the predicted secondary structure of At-*CHS* mRNA revealed that only one of the five major peaks in the trigger sequence corresponded to an extended length of self-complementarity (Supplemental Fig. S3). The positions of other peaks did not match predicted double-stranded fragments of At-*CHS* mRNA. In contrast, in multiple cases we detected associations between the distribution of peaks and the presence of primary or secondary ORFs (Fig. 3B,C; Supplemental Fig. S2). These data indicate that although presence of an ORF can decrease overall sRNA accumulation (Fig. 1), it can also determine the positions of sRNA peaks corresponding to the trigger sequence.

In addition to the quantitative effects discussed above, sequence translatability also has qualitative effects on sRNA production. The position of peaks of sRNA accumulation correlated with the position of predicted ORFs (Fig. 3; Supplemental Fig. S2). Furthermore, translatability can be influenced by several factors such as mRNA secondary structure and codon usage (Fig. 5; Supplemental Figs. S3, S4), both of which may cause ribosome stalling (Shoemaker and Green 2012). The 79-nt phasing of the peaks of sRNA produced from At-*CHS* (Fig. 3A,B) may therefore correspond to the positions of stalled ribosomes and/or the spaces in between stalled ribosomes that are accessible to the RNAi machinery. The facilitating role for SDE3 helicase during RDR6-mediated amplification of sRNA (Dalmay et al. 2000; Mourrain et al. 2000; Curaba and Chen 2008; Garcia et al. 2012) suggests a speculative but attractive explanation for the correlation between sRNA distribution and the presence of ORFs (Fig. 3; Supplemental Fig. S2). RNAi-mediated RNA decay and transitivity both involve RDR-mediated synthesis of dsRNA from single-stranded (ss) RNA templates that are likely perceived in the cell as aberrant (De Paoli et al. 2009; Gregory et al. 2009). Perhaps preferential accumulation of sRNA corresponding to ORFs in such aberrant mRNA (Fig. 3; Supplemental Fig. S2) could occur as a result of amplification involving the helicase activity of ribosomes (Takyar et al. 2005) working in conjunction with RDR6. Previously suggested competition between the translational machinery and RDRs for mRNA substrates (Gazzani et al. 2004; Gregory et al. 2009) could provide a sensitive mechanism for detecting aberrant RNA, exposing such RNA to RDR6 particularly during ribosome stalling. Amplification of sRNA corresponding to that aberrant RNA template could then possibly occur by a mechanism similar to that proposed by Garcia et al. (2012). Furthermore, the initially produced sRNA acting as translational repressors (Brodersen et al. 2008) could, in turn, increase ribosome stalling, RDR6 recruitment, and subsequent biogenesis of secondary sRNA, thereby resulting in clearly delineated peaks of sRNA accumulation. Whether or not a combination of inefficient translation and the helicase activity of ribosomes contribute to ihpRNA processing is an open question. However, the weak but significant correlation we observed between the distribution of sRNA corresponding to the *uid*A (*GUS*) target sequence in our experiments and the occurrence of rare codons in that fragment (Supplemental Fig. S4) serves as additional, indirect support for such a possibility.

In conclusion, our extensive analyses of sRNA associated with IR-PTGS in *Arabidopsis* provide insights into the interplay of IR-PTGS and endogenous pathways of sRNA biogenesis, as well as provide guidelines for designing ihpRNA constructs to optimize their efficiency for inducing PTGS. The similarities in patterns of sRNA production suggest that similar mechanisms underlie ihpRNA processing and RNAi-mediated decay involved in mRNA turnover. Finally, the profound effect of IR-PTGS on hc-siRNA, miRNAs, and ta-siRNAs may result in off-target effects that should be taken into consideration when interpreting RNA silencing experiments as well as implementing RNAi strategies for agricultural purposes.

## MATERIALS AND METHODS

Sequences from this article can be found in the GEO data libraries under accession number GSE38922 (http://www.ncbi.nlm.nih.gov/geo/query/acc.cgi?acc=GSE38922).

### Production of transgenic plants and assays for gene silencing

A 372-bp fragment of the At-*CHS* gene (Gene Bank # 03, bases 359–730) and a 342-bp fragment of the *uidA* gene (Gene Bank # M14641, bases 2430–2771) were fused together in different configurations and cloned into a modified version of binary vector pGSA1165 (http://chromdb.org/), in sense and antisense orientations (Fig. 1; Supplemental Fig. S1). Two arms of the inverted repeat in each construct were separated by intron-3 of the *PYRUVATE ORTHOPHOSPHATE DIKINASE* gene from *Flaveria trinervia* (Ft*PDK*; Gene Bank # X79095, bases 7890–8668) (Supplemental Fig. S1). In the two constructs containing the fused gene fragments adjacent to the promoter in the sense orientation, the fused fragments were cloned in frame with the ATG codon downstream from the 35S promoter in the vector backbone creating ORFs (Supplemental Fig. S1). (Details of vector modification and cloning are provided in Supplemental Materials and Methods.) All constructs were introduced into *Agrobacterium tumefaciens* strain LBA4404. Transgenic plants of *Arabidopsis thaliana* ecotype Wassilewskija (Ws-0; CS 915; TAIR, http://www.arabidopsis.org/) were produced using floral dip transformation (Clough and Bent 1998). Transient expression of GUS was induced in T_1_ plants using binary vector plasmid pTFS40 containing the *uidA* gene as described previously (Wroblewski et al. 2005). Six days after infiltration two to three leaves from each plant were pooled together for protein extraction and quantitative evaluation of GUS activity using 4-methylumbelliferyl-β-D-glucuronide (MUG)-based fluorimetrical assay (Jefferson et al. 1987) in microtiter plates with an Analyst AD instrument (Molecular Devices, http://www.moleculardevices.com/) at an excitation wavelength of 360 nm and an emission wavelength of 460 nm. Induction of transient GUS expression and GUS activity measurement were performed three times in each T_1_ plant and in three nontransgenic Ws-0 plants as a control. Data presented in Figure 1B show average values obtained from these three replicates, were standardized to total protein content in the extracts, and comprise over 700 measurements. The same values were also used for correlation analysis. Silencing of At-*CHS* was visually evaluated based on the level of anthocyanin production in T_2_ plants. The T_2_ families were randomized in a growth chamber and grown in three replicas under stressful conditions: light intensity of ∼300 μE, 14 h light/10 h dark photoperiod, 15°C during the day and 5°C during the night. After 4 wk, each plant was assigned to one of four phenotypic categories as shown in Figure 1C: red—anthocyanin levels similar to wild-type, no silencing; red-yellow—coloration strong but weaker than the wild-type, weak silencing; yellow-green—only traces of anthocyanin present in all leaves, strong silencing; and green—no anthocyanin detected, complete silencing. The efficacy of silencing in each T_2_ family was computed as the number of completely green plants plus half of all the plants with only traces of anthocyanin divided by the total number of plants analyzed for that family. At least 48 individual plants were evaluated per each T_2_ family for a total of over 17,000 plants. (For more details on plant assays and data analyses see Supplemental Materials and Methods.)

### Construction of sRNA libraries and sequencing

Each sRNA library was made from RNA isolated from 120 plants grown for 4 wk under stressful conditions as described above and pooled together. Ten silenced plants (lacking anthocyanin) from each of 12 selected T_2_ families per construct (indicated with a white rectangle in Fig. 1C) were harvested and used for total RNA extraction using Trizol (Invitrogen Inc.; http://www.invitrogen.com/). The libraries were made using Small RNA Sample Prep Kits (Illumina Inc.; http://www.illumina.com/) following the manufacturer's protocol. Gel-purified fractions of sRNA from 10 μg total RNA were used as an input material. Small RNAs were ligated to Illumina-supplied 5′ and 3′ adapters. The products of amplification corresponding to 18- to 30-nt long RNAs were excised from Novex 6% TBE PAGE gel (Invitrogen Inc.; http://www.invitrogen.com/) and sequenced on the Genome Analyzer II using 41 (Libs 0, 5, and 6) or 45 (Libs 1–4) cycles.

### Analysis of sRNAs

Sequences were extracted from the GAII image files using the standard Illumina pipeline (http://code.google.com/p/atgc-illumina/source/browse/#svn/trunk). Details related to sig2 base-calling are available at https://code.google.com/p/atgc-illumina/. A custom Perl script was used to identify and trim adapter sequences. Because no substantial differences were found between libraries after alignment to a set of 40 genomic reference loci, no further normalization was performed. Reads lacking adapter sequences or shorter than 18 nt after trimming were removed from further analysis. Five hundred thousand sequences from each library were mapped to four query sequences corresponding to the four different constructs using BLAST (Altschul et al. 1990) with a word size of 10 and expectation value of 0.1. A custom Python script (http://code.google.com/p/xuhu-rwm-blast/wiki/Blastcovct) was used to generate tables with coverage values per nucleotide of query sequence. Significance of the position-dependent differences in sRNA production among the four constructs was determined using the χ^2^ test and the cumulative values of sRNA representations for each fragment, At-*CHS* or *uidA*. Correlation between abundance of sRNAs in peaks and their position in an ihpRNA (Fig. 2) was calculated using the distance from loop (nt) and sRNA abundance in ten peaks standardized to the mean. Analyses of endogenous sRNAs were performed using 1 million randomly selected sequences from each library and the reference sequence of five *Arabidopsis* chromosomes (http://www.ncbi.nlm.nih.gov). The alignments were created using CLC Genomics Workbench 4.9 (http://www.clcbio.com/) and the results, retrieved as SAM formatted files, were filtered using a custom Python script (http://code.google.com/p/xuhu-rwm-blast/wiki/Blastcovct) that also calculated and compared the coverage of 20–21 and 23–24 sequences from each library over each of the five *Arabidopsis* chromosomes.

### Calculation of codon usage, prediction of ORFs, and determination of mRNA secondary structures

For *A. thaliana* and *Petunia hybrida*, we used a codon usage table calculated for all coding sequences (http://www.kazusa.or.jp/). The relative codon frequency (RCF; rarity) was calculated using an approach similar to that published previously (Clarke and Clark 2008). For the *j*th codon of the *i*th amino acid with n synonymous codons, the algorithm calculates the RCF factor by which the actual codon usage frequency (*X*_*ij*_) differs from the average frequency of synonymous codons for a particular amino acid (*X*_avg,*i*_):


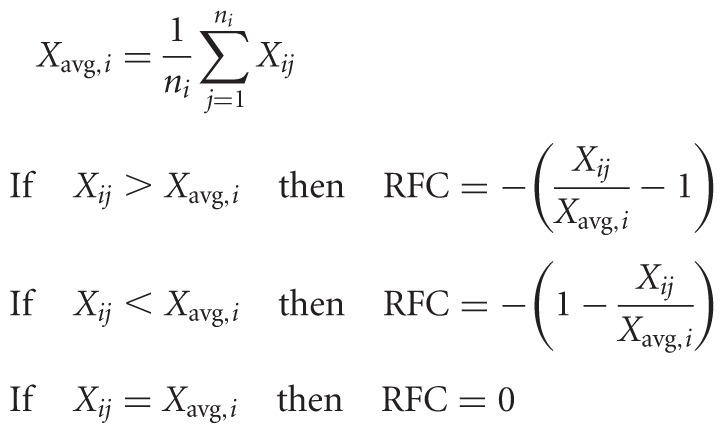


For clarity of display we multiplied the RFC by negative 1, and therefore the less frequent a particular codon among all synonymous codons, the higher the value assigned to it and *vice versa*. The relative abundance of rare codons presented in Figure 4 shows the average values for RCF calculated using for 20-codons long sliding (by one codon) windows. The *X*_*ij*_ values were obtained from the Kazusa DB. Presence of ORFs was predicted using a publicly available online tool (http://www.ncbi.nlm.nih.gov/projects/gorf/). Correlations between codon frequency and sRNA production were analyzed using RFC value for each codon and the average abundance of sRNA over the three nucleotides comprising that codon using Microsoft Excel. Significance of these correlations was determined using critical *P* values in the two-tailed test. Secondary mRNA structures were determined by Mfold (Zuker 2003) with default settings. Most calculations, statistical analyses and data displays were carried out using Microsoft Excel.

### Construction of polyA RNA libraries and analysis of RNA-Seq data

RNA-Seq libraries were made using poly-A RNA purified from total RNA preparations obtained for sRNA libraries (described above) using Dynabeads oligo(dT)_25_ and manufacturer's protocol (Dynabeads mRNA direct kit; http://www.lifetechnologies.com). The cDNA was synthesized and fragmented to obtain 250-bp insert size using Covaris (http://covarisinc.com) according to a previously published protocol (Zhong et al. 2011) except for PCR enrichment. Each library was barcoded using Illumina-compatible NEXTflex DNA Barcodex (http://www.biooscientific.com). Individual libraries were pooled and sequenced using Illumina HiSeq 2000 sequencer to obtain 100 × 100 nt paired-end reads. The raw Illumina sequence reads were processed for barcode removal (CASAVA v1.8), adapter removal (Scythe program), filtered and trimmed for quality (allPrep script by M. Lieberman, University of California Davis; http://comailab.genomecenter.ucdavis.edu/index.php/Barcoded_data_preparation_tools). Nine million randomly selected reads from each library were used for further analysis. Alignment to *Arabidopsis* rRNA-5S-encoding sequences, thirteen reference sequences used previously for transcript normalization (Czechowski et al. 2005), and to the At-*CHS* mRNA sequence was performed using CLC Genomics Workbench (http://www.clcbio.com).

## SUPPLEMENTAL MATERIAL

Supplemental material is available for this article.

## Supplementary Material

Supplemental Material
