# Supplementary material for: Distinctive profiles of small RNA couple inverted repeat-induced post-transcriptional gene silencing with endogenous RNA silencing pathways in Arabidopsis
Source: RNA. 2014 Dec;20(12):1987–99. doi: 10.1261/rna.046532.114 (PMC4238362; doi:10.1261/rna.046532.114)
Supplement: Supplemental Material [file supp_20_12_1987__index.html]

Distinctive profiles of small RNA couple inverted repeat-induced post-transcriptional gene silencing with endogenous RNA silencing pathways in Arabidopsis — Distinctive profiles of small RNA couple inverted repeat-induced post-transcriptional gene silencing with endogenous RNA silencing pathways in Arabidopsis — Supplemental Material 

# Distinctive profiles of small RNA couple inverted repeat-induced post-transcriptional gene silencing with endogenous RNA silencing pathways in *Arabidopsis*

## Supplemental Material

**Files in this Data Supplement:**

- Supp Material.pdf
- Supp Table S3.pdf
