# Supplementary material for: Distinctive profiles of small RNA couple inverted repeat-induced post-transcriptional gene silencing with endogenous RNA silencing pathways in Arabidopsis
Source: RNA. 2014 Dec;20(12):1987–99. doi: 10.1261/rna.046532.114 (PMC4238362; doi:10.1261/rna.046532.114)
Supplement: Supplemental Material [file supp_046532.114_Supp_Table_S3.pdf]

Supplemental Table S3. Total number of reads and RPKM (reads per kbp per million) for 13 reference sequences in 14 RNA-Seq libraries. Reference genes were previously used for transcript normalization (Czechowski et al., 2005) were selected for level of expression comparable

Locus	Transcript length	ESeq 0 Total number of reads	Eseq 0 - RPKM	RSeq 0 Total number of reads	RSeq 0 - RPKM	ESeq 1 Total number of reads	Eseq 1 - RPKM
**AT4G26410**	1123	176	21.056	77	10.156	142	17.204
**AT2G28390**	2170	314	19.441	458	31.263	261	16.364
**AT5G46630**	1757	458	35.022	256	21.582	416	32.214
**AT2G32170**	2365	167	9.487	196	12.276	189	10.873
**PP2A-1**	1301	290	29.948	252	28.691	223	23.321
**AT3G12590**	4306	141	4.399	154	5.297	122	3.855
**AT1G07920**	1783	1375	103.611	1069	88.807	1318	100.573
**AT4G33380**	1427	387	36.437	311	32.282	375	35.754
**AT4G34270**	1254	195	20.892	260	30.711	193	20.94
**YLS8**	715	1383	259.878	741	153.509	1507	286.765
**PTB1**	1511	142	12.626	260	25.488	170	15.307
**PP2AA3**	2128	341	21.53	245	17.054	346	22.122
**AT5G60390**	1764	1631	124.225	375	31.489	557	42.961

Locus	RSeq 1 Total number of reads	RSeq 1 - RPKM	ESeq 2 Total number of reads	Eseq 2 - RPKM	RSeq 2 Total number of reads	RSeq 2 - RPKM	ESeq 3 Total number of reads	Eseq 3 - RPKM
**AT4G26410**	74	10.324	46	5.756	54	6.849	120	18.355
**AT2G28390**	288	20.794	611	39.566	471	30.918	204	16.148
**AT5G46630**	311	27.732	246	19.675	392	31.78	379	37.052
**AT2G32170**	211	13.978	264	15.686	194	11.685	148	10.749
**PP2A-1**	241	29.023	323	34.888	223	24.416	177	23.369
**AT3G12590**	115	4.184	110	3.59	174	5.756	88	3.51
**AT1G07920**	1004	88.223	871	68.645	990	79.091	966	93.061
**AT4G33380**	285	31.291	313	30.822	329	32.841	272	32.741
**AT4G34270**	183	22.864	292	32.721	219	24.877	137	18.766
**YLS8**	1210	265.142	768	150.939	1127	224.523	1225	294.289
**PTB1**	188	19.494	194	18.042	171	16.12	167	18.984
**PP2AA3**	288	21.204	219	14.462	344	23.027	277	22.359
**AT5G60390**	474	42.1	267	21.27	435	35.126	422	41.092

Locus	RSeq 3 Total number of reads	RSeq 3 - RPKM	ESeq 4 Total number of reads	Eseq 4 - RPKM	RSeq 4 Total number of reads	RSeq 4 - RPKM	ESeq 5 Total number of reads	Eseq 5 - RPKM
**AT4G26410**	38	4.885	113	16.304	78	10.077	103	15.204
**AT2G28390**	502	33.394	242	18.07	468	31.289	226	17.264
**AT5G46630**	207	17.007	392	36.15	252	20.808	353	33.304
**AT2G32170**	196	11.963	137	9.386	241	14.784	156	10.934
**PP2A-1**	233	25.852	218	27.15	207	23.084	220	28.031
**AT3G12590**	155	5.196	134	5.042	139	4.683	137	5.274
**AT1G07920**	873	70.678	952	86.513	865	70.384	953	88.601
**AT4G33380**	279	28.223	274	31.111	339	34.466	242	28.112
**AT4G34270**	288	33.152	198	25.584	225	26.031	190	25.116
**YLS8**	1059	213.801	1234	279.643	1028	208.591	1120	259.661
**PTB1**	215	20.54	143	15.334	202	19.395	191	20.954
**PP2AA3**	231	15.67	295	22.462	87	5.931	325	25.317
**AT5G60390**	0	0	390	35.823	366	30.102	436	40.972

Locus	RSeq 5 Total number of reads	RSeq 5 - RPKM	ESeq 6 Total number of reads	Eseq 6 - RPKM	RSeq 6 Total number of reads	RSeq 6 - RPKM
**AT4G26410**	38	4.773	124	17.008	62	8.135
**AT2G28390**	537	34.904	226	16.042	436	29.607
**AT5G46630**	278	22.317	374	32.788	239	20.045
**AT2G32170**	199	11.868	174	11.333	213	13.271
**PP2A-1**	196	21.249	230	27.231	275	31.148
**AT3G12590**	172	5.634	108	3.863	156	5.339
**AT1G07920**	990	78.316	1069	92.35	810	66.943
**AT4G33380**	318	31.432	322	34.757	354	36.555
**AT4G34270**	178	20.021	195	23.952	270	31.728
**YLS8**	1081	213.248	1151	247.96	874	180.126
**PTB1**	215	20.07	167	17.024	255	24.868
**PP2AA3**	320	21.21	312	22.584	198	13.711
**AT5G60390**	385	30.784	499	43.573	357	29.822

**Aver. Eseq 0 - 6:**	46.27607692	
		p= 0.00466
**Aver. Rseq 0 - 6:**	39.77007692	1-Tailed T-test
**Percent of change:**	16.35903298	
