# Supplementary material for: Distinctive profiles of small RNA couple inverted repeat-induced post-transcriptional gene silencing with endogenous RNA silencing pathways in Arabidopsis
Source: RNA. 2014 Dec;20(12):1987–99. doi: 10.1261/rna.046532.114 (PMC4238362; doi:10.1261/rna.046532.114)
Supplement: Supplemental Material [file supp_046532.114_Supp_Material.pdf]

Distinctive profiles of small RNA couple inverted repeat-induced posttranscriptional gene silencing with endogenous RNA silencing pathways in Arabidopsis

SUPPLEMENTAL MATERIALS:
Supplemental Figures S1 to S7
Supplemental Material and Methods
Supplemental Tables S1 and S2
Supplemental reference

SUPPLEMENTAL FIGURES

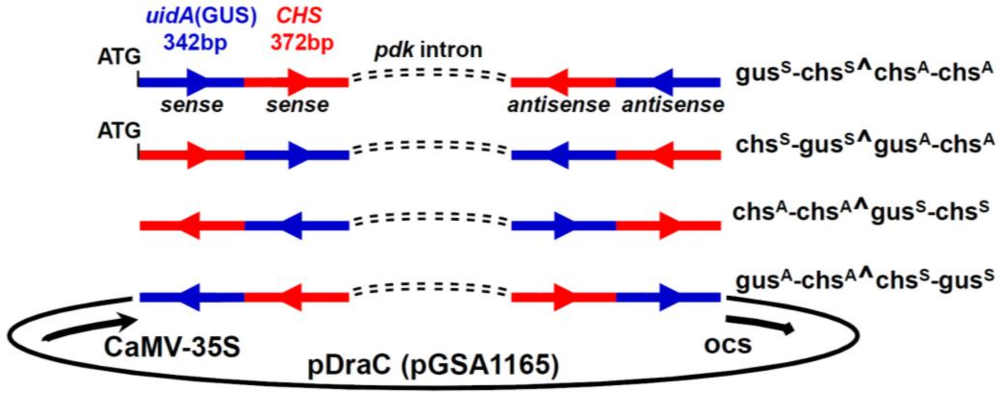

SUPPLEMENTAL FIGURE S1. Different ihpRNA constructs used to test the effect of coding sequence orientation on the efficiency of IR-PTGS. The gus^S-chs^S^chs^A-chs^A and chs^S-gus^S^gus^A-chs^A fragments of the *uidA* (GUS) and At-*CHS* sequences, respectively, were introduced to the vector pDraC in the sense orientation. Therefore the first arm of the inverted repeat in these two constructs contains an ORF initiated by the ATG codon immediately preceding the cloning site adjacent to the CaMV-35 promoter. ^{S} and ^{A} in the construct's name = sense and antisense orientation, respectively; ^ = the position of the intron.

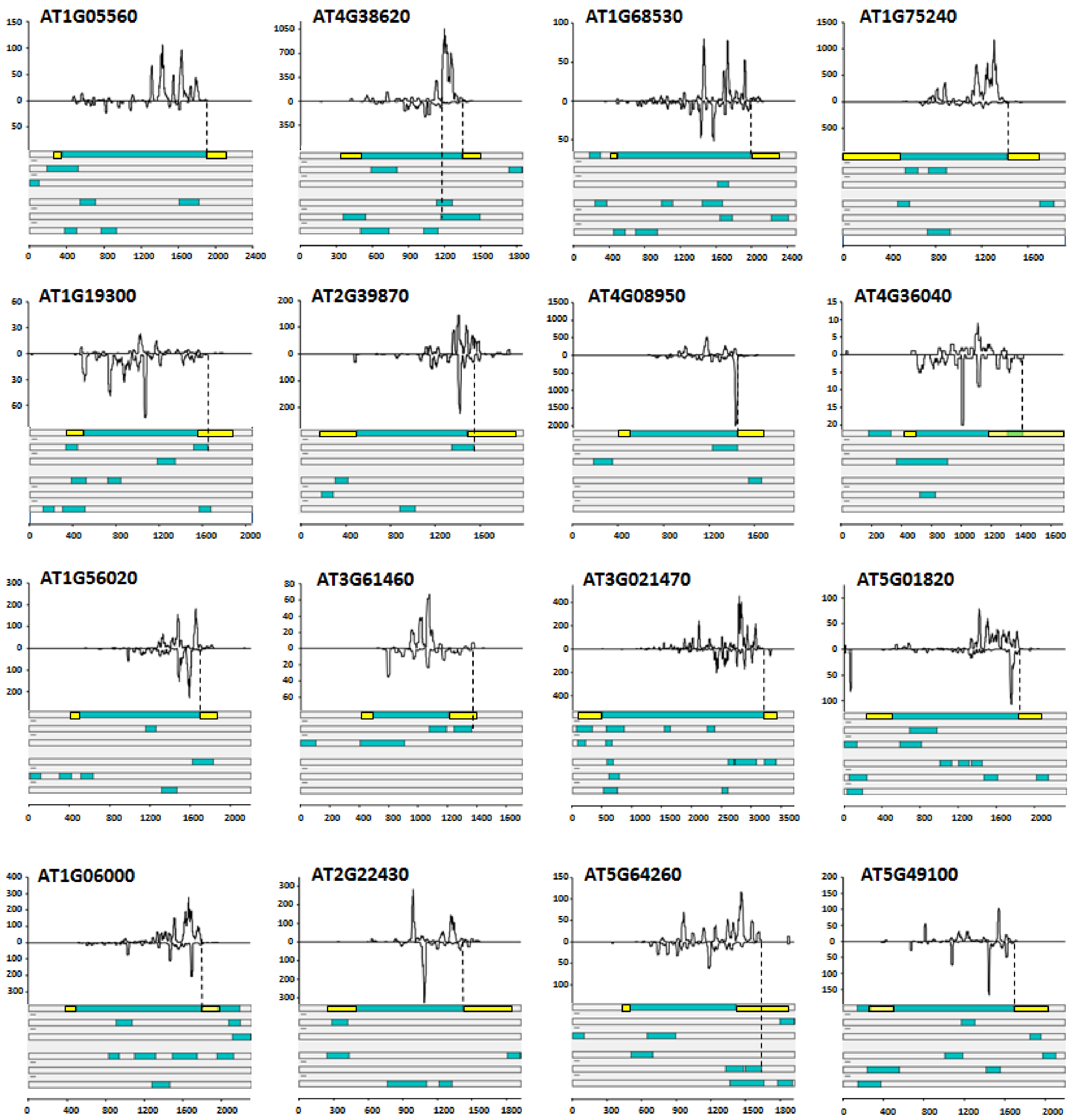

SUPPLEMENTAL FIGURE S2. Association of sRNA production with predicted ORFs in six putative reading frames among 16 genes affected by uncapped mRNA decay (Gregory et al. 2009). Small RNA is produced predominantly from sequences containing primary or secondary ORFs in the (+) or (-) strand as opposed to being produced from the sequences comprising 3'UTRs. In a few cases in which sRNAs corresponded to 3'UTRs (*e.g.*, AT4G36040, AT3G61460, AT5G64260) their positions nearly exactly matched the secondary ORFs predicted in that fragment. Blue indicates ORFs; yellow indicates UTRs next to the main ORF. The presence of ORFs was determined by ORF Finder (http://www.ncbi.nlm.nih.gov/projects/gorf/orfig.cgi) with default settings.

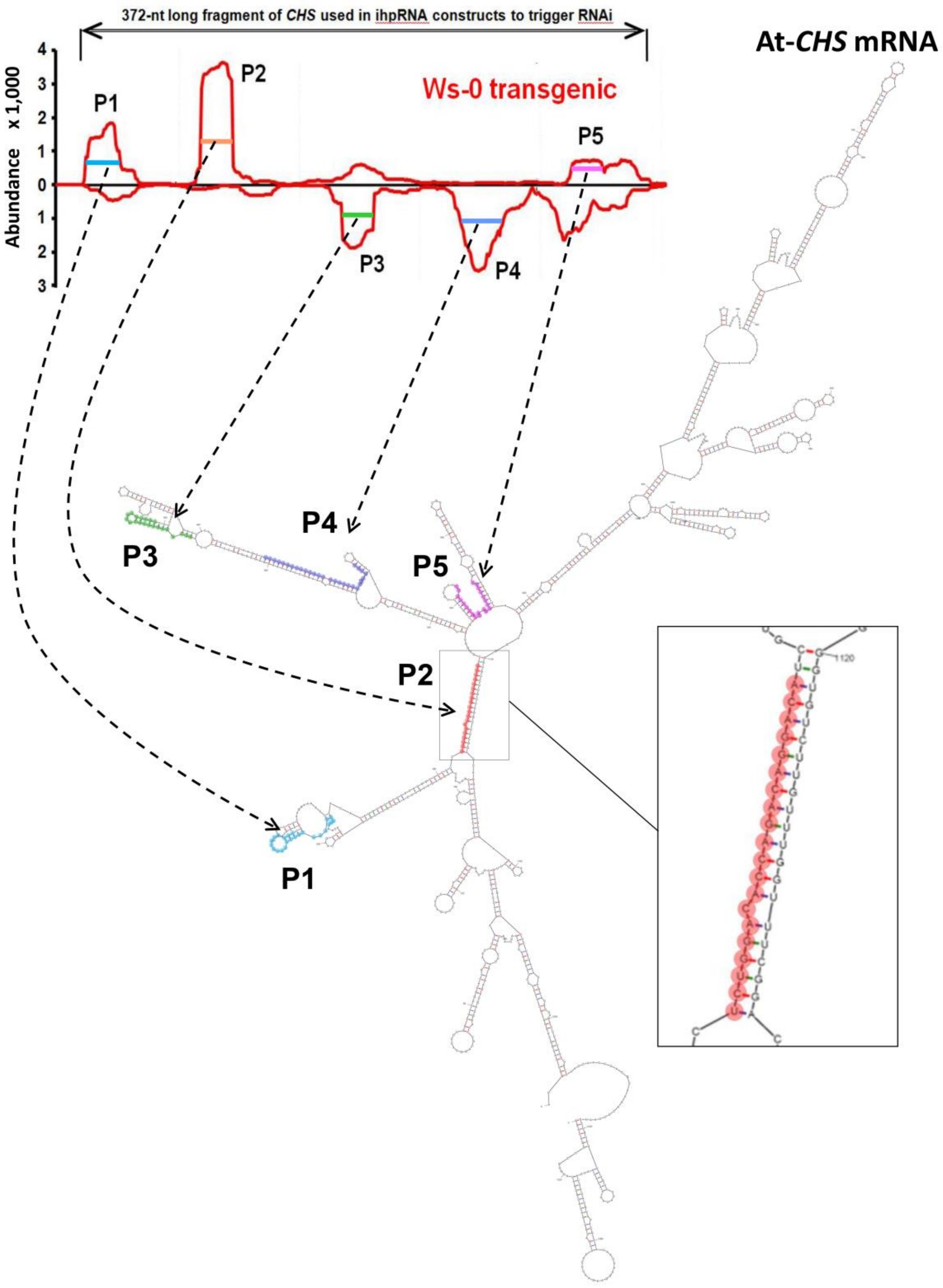

SUPPLEMENTAL FIGURE S3. Positions of the sRNA peaks mapped to the secondary structure predicted for the entire sequence of At-*CHS* mRNA. Small RNAs accumulated in the P2 peak were produced from a fragment of the At-*CHS* mRNA that contains dsRNA with one mismatch and resembles pre-microRNA (Lee at al. 2004).

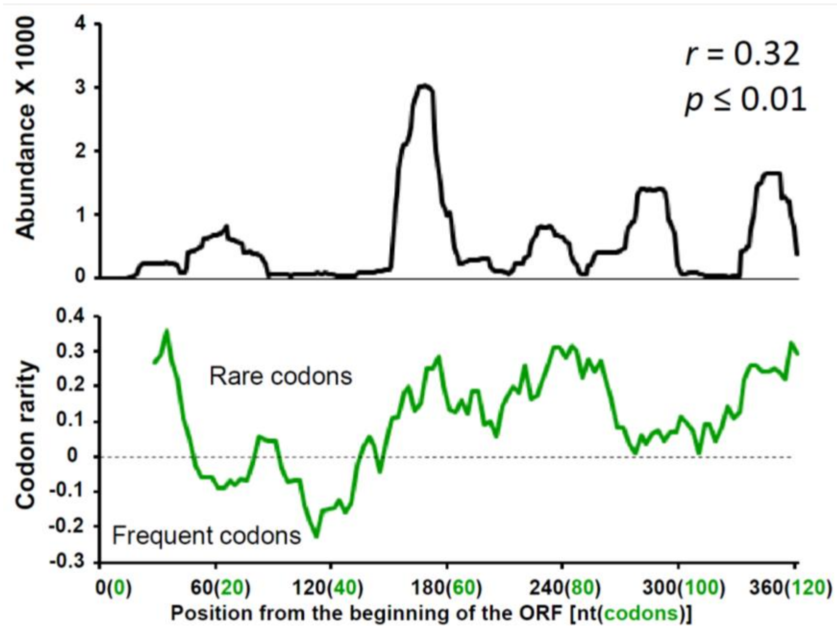

SUPPLEMENTAL FIGURE S4. Abundance of sRNA mapped to the fragment of *uidA* (top panel) and the frequency of rare codons (bottom panel) in this sequence. Production of sRNAs shows weak but significant correlation with the fragment of the sequence containing elevated abundance of rare codons. Abundance is shown as the number of matches per nt per million reads. Codon rarity was calculated using a sliding window of ten codons using an algorithm adapted from (Clarke Clark 2008).

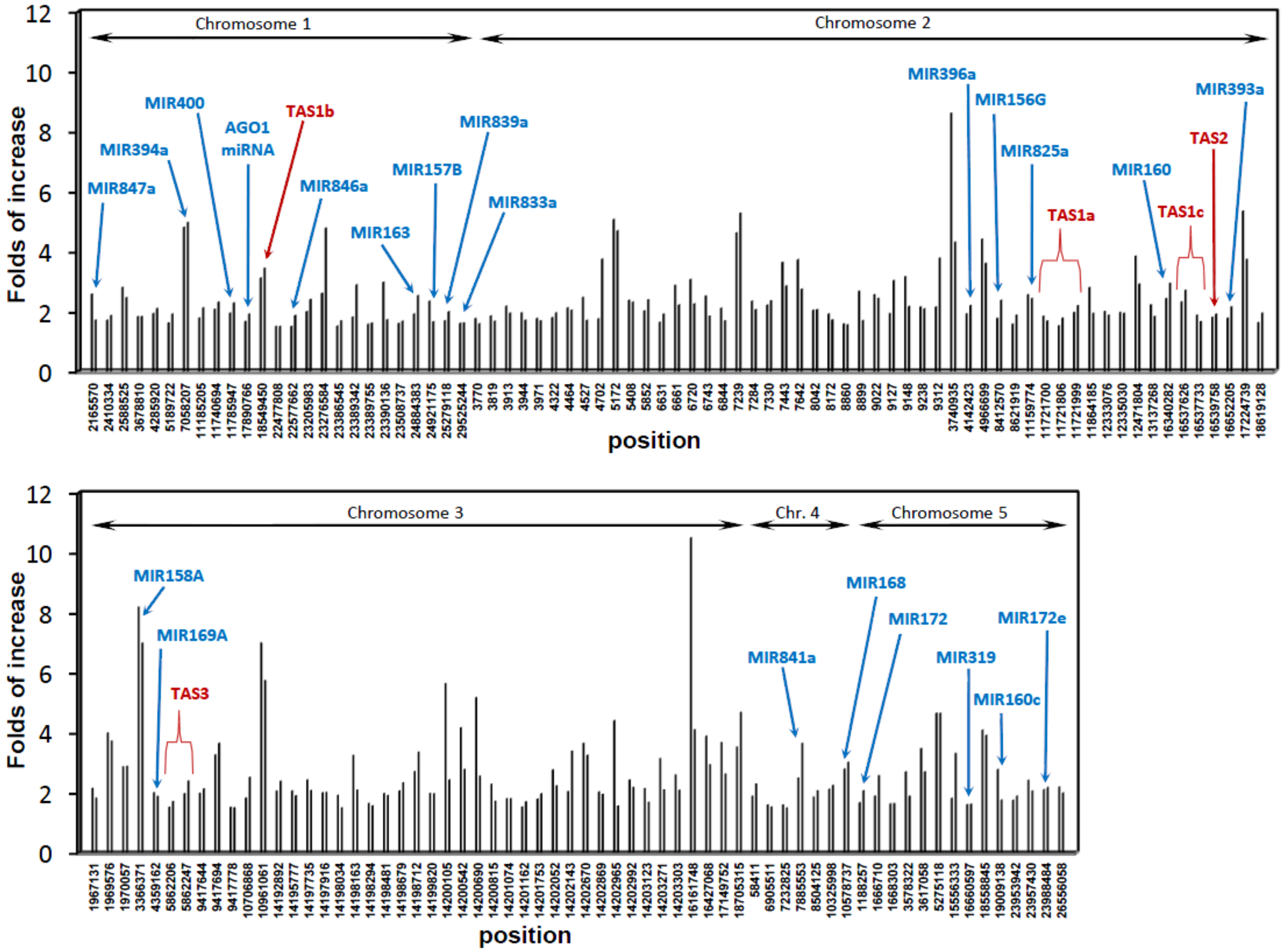

SUPPLEMENTAL FIGURE S5. Genomic loci for which corresponding 20- to 21-nt long sRNAs were increased in strongly silenced plants by at least 1.5 fold. The two bars for each locus represent the fold increases in comparisons of Lib 0 with Lib 5 and Lib 0 with Lib 6. Red = loci encoding tasiRNA, blue = loci encoding miRNAs.

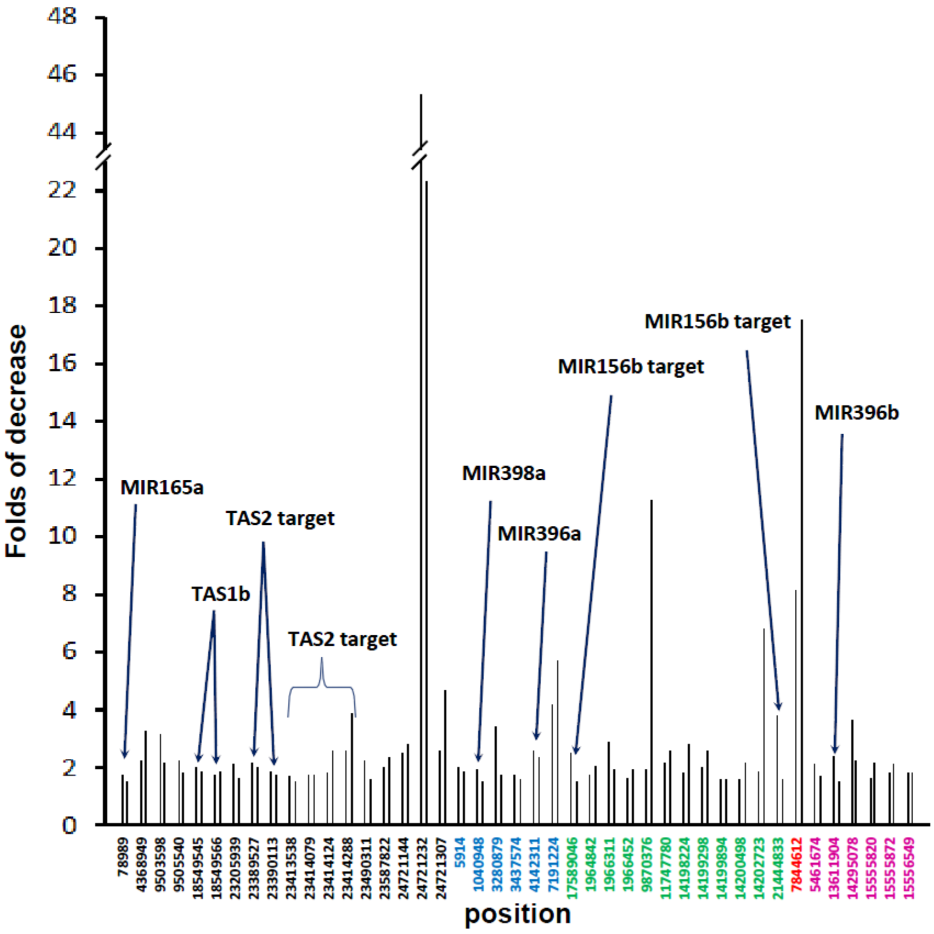

SUPPLEMENTAL FIGURE S6. Genomic loci for which corresponding 20- to 21-nt long sRNAs were decreased in strongly silenced plants by at least 1.5 fold. The two bars for each locus represent fold decreases in comparisons of Lib 0 and Lib 5, and Lib 0 and Lib 6, respectively. Black, blue, green, red, and purple indicate positions of these loci in chromosomes 1 through 5, respectively.

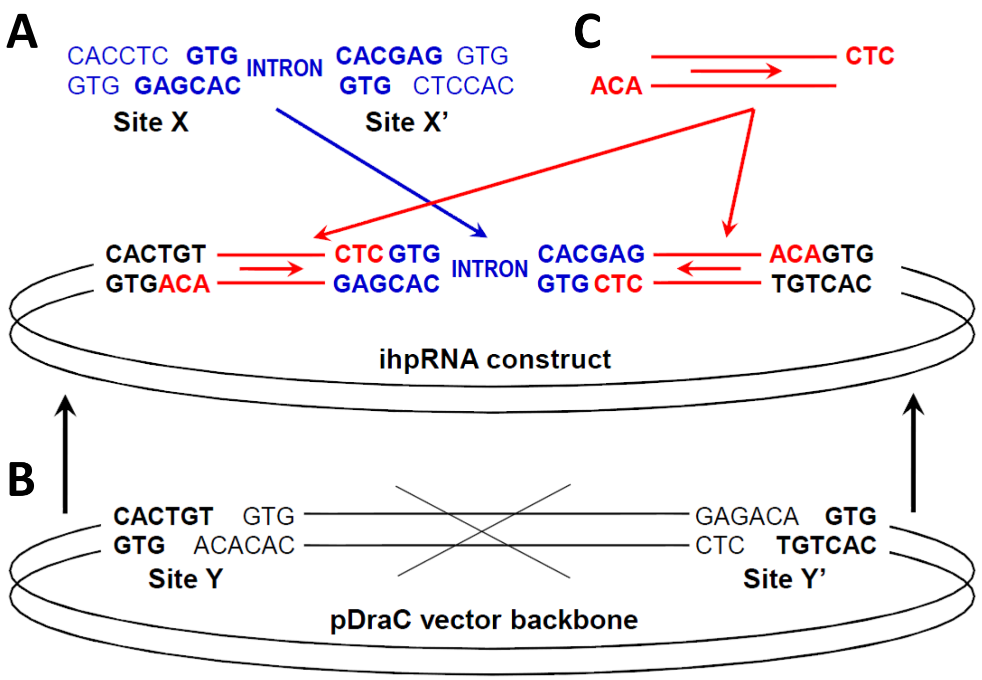

SUPPLEMENTAL FIGURE S7. Production of ihpRNA constructs using the pDraC vector. (***A***) Two *Dra*III restriction sites flank intron-3 of the pdk gene. Cleavage with *Dra*III results in the production of an intron fragment flanked by two identical 3-bp overhangs. (***B***) Cleavage at two *Dra*III restriction sites (located between the CaMV-35S promoter and the terminator in the pDraC vector backbone) results in a linear vector molecule flanked by two identical 3-bp overhangs (different from those flanking the intron). (***C***) The fragment of the gene targeted for silencing is amplified with primers that introduce two different flanking *Sfi*I restriction sites. Cleavage with *Sfi*I produces one sticky end compatible with sites X and X' and a second one compatible with sites Y and Y'. During ligation the insert fragment is incorporated into the ihpRNA construct twice, once in the sense and once in the antisense orientation.

SUPPLEMENTAL MATERIALS AND METHODS

Production of ihpRNA constructs. All constructs for plant transformation (Supplemental Fig. S1) were made in a modified binary vector pGSA1165 (http://chromdb.org/). All restriction endonucleases used in this work were from New England Biolabs (http://www.neb.com/). T4 Ligase and TOPO pCR 2.1 cloning kit was from Invitrogen (http://www.invitrogen.com/). PCR amplifications were performed using Advantage HF polymerase (Clontech, Mountain View, CA) according to the manufacturer's protocol. DNA purified by preparative gel electrophoresis was recovered from 1% agarose gels using QIAEX II Gel Extraction Kits (Qiagen; http://www.qiagen.com/).

Construction of pDraC Vector Backbone:

The pDraC plasmid was made by introducing two symmetrical *Dra*III restriction sites (Site Y and Site Y', Supplementary Figure S7) into the pGSA1165 binary vector (http://www.chromdb.org) next to the *Nco*I and *Bam*HI sites between the CaMV-35S promoter and ocs3' transcription terminator. *Nco*I/*Bam*HI digested pGSA1165 was ligated to *Nco*I/*Bam*HI-digested *GFP* fragment (linker) amplified by PCR using NDLF and BDLR primers (Supplemental Table S1). Next, the pDraC

plasmid was cleaved with *Dra*III and purified to produce the pDraC vector backbone. This vector backbone was used in all following cloning steps.

Production of the Intron-Containing Fragment:

We made three intron-containing fragments and used them to produce four different ihpRNA assemblies shown in Supplementary Figure S1: (1) ‘intron’, (2) ‘gus^S-intron-gus^A’ and (3) ‘chs^S-intron-chs^A’(^{S} and ^{A} stand for the orientation of the fragment; sense and antisense respectively). All three fragments were flanked by two symmetrical *Dra*III restriction sites (Site X and Site X’, Supplementary Figure S7a). The ‘intron’ contained the sole intron, Intron-3, of the *PYRUVATE ORTHOPHOSPHATE DIKINASE* gene from *Flaveria trinervia* (Ft*PDK*) and was produced by PCR amplification of the Ft*PDK* gene fragment (Gene Bank # X79095, bases 7890 to 8668) using DIntF and DIntR primers (Supplemental Table S1) introducing *Dra*III sites (Site X and Site X’; Supplemental Fig. S7) on each end of the molecule. The amplification product was introduced into TOPO pCR 2.1 TA vector; the sequence-verified clone was cleaved using *Dra*III, purified and used in the following cloning steps. The ‘gus^S-intron-gus^A’ fragment contained in addition to Intron-3 an IR of the 342-bp long *uidA* gene fragment (Gene Bank # M14641, bases 2430 to 2771) and the ‘chs^S-intron-chs^A’ fragment contained an IR of the 372-bp long At*CHS* gene fragment (Gene Bank # Y18603, bases 359 to 730). To make the ‘gus^S-intron-gus^A fragment, the fragment of the *uidA* gene was amplified by PCR using DGUSF and SGUSR primers (Supplemental Table S1) designed to introduce a *Dra*III cleavage site on one end of the molecule and a *Sfi*I site on the other end. The product of amplification was digested with *Sfi*I and purified. The sticky end produced after cleavage digestion with *Sfi*I was compatible with both of the sticky ends (Site X and Site X’) present in the ‘intron’ fragment (compare to Supplemental Fig. S7). Next this *uidA* gene fragment was ligated to the ‘intron’ fragment using a molar ratio of 2 to 1 respecitively. The ligation product was then cloned into the TOPO pCR 2.1 vector. This ligation resulted in a chimeric molecule containing the Intron-3 flanked on each end by symmetrical *uidA* fragments, each oriented as an IR with respect to the other. Ligation of the compatible *Dra*III (from the ‘intron’) and *Sfi* I (from the *uidA* fragment) sticky ends inactivated for future cleavage the *Dra*III restriction sites. Instead two new symmetrical sites, Site X and Site X’, were introduced into the molecule at each end, along with the two *uidA* gene fragments. The verified clone in the TOPO pCR 2.1 vector was cleaved with *Dra*III, the ‘gus^S-intron-gus^A’ fragment was purified and used in subsequent cloning steps. The ‘chs-intron-shc’ fragment was prepared in a similar manner as ‘gus^S-intron-gus^A’ except that instead of the 342 bp fragment of the *uidA* gene, we used a 372 bp fragment of the At*CHS* gene. Two primers (DCHSF and SCHSR, Supplemental Table S1) were used to amplify the At*CHS* gene fragment prior to its fusion to the intron-encoding fragment of DNA. Both the fragment of the *GUS* gene and the fragment of At*CHS* were designed to be in frame with the two codons immediately preceding the intron sequence. The resulting clone was digested with *Dra*III and the ‘chs^S-intron-chs^A’ fragment was purified by preparative electrophoresis and used in subsequent cloning steps.

Production of Inverted Repeat (IR) Clones Used To Generate Transgenic Plants:

IR structures present in the four constructs in Supplementary Figure S1 were produced via a four-molecule ligation reaction involving one molecule of the pDraC vector backbone, one molecule of the intron-containing fragment and two molecules of an insert (a fragment of the gene targeted for silencing) (Supplemental Fig. S7). Accordingly the clone chs^S-gus^S^gus^A-chs^A was made by assembling the pDraC vector backbone with the ‘gus^S-intron-gus^A’ intron-containing fragment and two 372-bp insert fragments of the At*CHS* gene spanning the same fragment as used to make ‘chs^S-intron-chs^A’. The insert was amplified by PCR using the SCHSF and SCHSR primers (Supplemental Table

S1) designed to introduce *Sfi*I restriction site on each end of the molecule. One *Sfi*I restriction site was compatible with Y and Y' *Dra*III sites flanking the vector backbone and the second one was compatible with X and X' *Dra*III sites flanking the intron-containing fragment. The position of the *Sfi*I restriction sites kept the At*CHS* gene fragment in frame with the ATG codon following the promoter in the vector backbone and the *uidA* gene fragment in 'gus^S-intron-gus^A'. The gus^S-chs^S^sch^A-gus^A clone was made in similar way by assembling the pDraC vector backbone with the 'chs^S-intron-chs^A' intron-containing fragment and two 342-bp insert fragments of the *uidA* gene spanning the same fragment as used to make 'gus^S-intron-gus^A'. The insert was amplified using two primers (SGUSF and SGUSR, Supplemental Table S1) and designed to produce a fragment of DNA that would be, after assembly, in frame with the ATG codon following the promoter in the vector backbone and the 'chs^S-intron-chs^A' fragment. Next, chs^A-gus^A^gus^S-chs^S was made by ligation of the pDraC backbone with the "intron' fragment and two inserts produced by PCR amplification of the chimeric gus-chs sequence from the gus^S-chs^S^chs^A-gus^A clone using SgusF and SchsR primers (Supplemental Table S1). Finally, gus^A-chs^A^chs^S-gus^S was made by ligation of the pDraC backbone with the 'intron' fragment and two inserts produced by amplification of the chimeric chs-gus sequence from chs^S-gus^S^gus^A-chs^A using SchsF and SgusR primers (Supplemental Table S1). Orientation of the intron in all four clones was determined by cleavage with *Bgl*II.

Analyses of transgenic plants. Transgenic plants of *A. thaliana* ecotype Ws-0 (CS 915; TAIR, http://www.arabidopsis.org/) were produced by floral dip transformation (42) using *Agrobacterium tumefaciens* strain LBA4404. Transgenic plants were selected using 50 mg/L of kanamycin sulfate (K4378; Sigma-Aldrich). The activity of β-glucuronidase was quantitatively evaluated (Jefferson et al. 1987) in leaves of plants that had been grown in a growth chamber (16/8h photoperiod, 22°C day and 18°C night, 60 percent humidity). Transient expression of *uidA* (*GUS*) was induced according to a previously described protocol (Wroblewski et al. 2005) using. *A. tumefaciens* strain C58 carrying binary plasmid pTFS40. Fluorescence of 4-methylumbelliferone (4MU) was measured in micro-titer plates with an Analyst AD instrument (Molecular Devices, http://www.moleculardevices.com/) at an excitation wavelength of 360 nm and an emission wavelength of 460 nm. Transient expression assays were performed three times on each of 239 T_1 plants and the data presented in Fig. 1B shows the average values. The effect of At*CHS* silencing was evaluated based on differences in leaf color related to anthocyanin production of plants grown under stressful high light conditions. Seeds of each T_2 family were grown in light intensity of ~300 μE, 14 h light/10h dark photoperiod at15°C during the day and 5°C during the night in three randomized replications. After four weeks each plant was assigned to one of four phenotypic categories as shown in Figure 1c: red – presence of anthocyanin similar to the wild type, no silencing, red-yellow – coloration weaker compared to the wild type, weak silencing; yellow-green – traces of anthocyanin present in all leaves, and green – no anthocyanin detected, totally green plants (as shown in Fig. 1C). The efficacy of silencing in each T_2 family was established as the number of completely green plants plus half of all the plants with only traces of anthocyanin (green-yellow; Figure 1c) divided by the total number of plants analyzed for that family. Unpaired T tests were performed on the pooled "efficacy of silencing" data to determine significance of differences in the efficacy of At*CHS* silencing between constructs. At least 48 individual plants were evaluated per each T_2 family (over 17,000 plants total).

Supplemental Table S1. Primers used to PCR-amplify components of the vector and ihpRNA constructs. Restriction sites are indicated in bold.

Name	Sequence 5' to 3' (restriction sites shown in bold)	Restriction site	Sequence amplified	Construct made
**NDLF**	AT**CCATGGCACTGTGTG**CAACACTTGTCACTACTTTCTCTT	*Nco*I, *Dra*III	*GFP*	**pDraC (linker)**
**BDLR**	AT**GGATCCACTGTGTG**TTAAAGCTCATCATGTTTGTATAG	*Bam*HI, *Dra*III		
**DIntF**	**CACCTCGTG**TTCCTTGGTAAGGAAATAATTATTTTC	*Dra*III	Ft*PDK*	**intron**
**DIntR**	**CACCTCGTG**TCCCAACTGTAATCAATCCAAATG	*Dra*III		
**DGUSF**	**CACCTCGTG**GGCCAACTCCTACCGTACCTC	*Dra*III	*uidA*	**gus^{S}-intron-gus^{A}**
**SGUSR**[1]	AT**GGCCTGAGTGGCC**GTCGAGTTTACGCGTTGCTTCC	*Sfi*I		
**DCHSF**	**CACCTCGTG**CATTCGGAAACGTCACATGCATCT	*Dra*III	At*CHS*	**chs^{S}-intron-shc^{A}**
**SCHSR**[1]	AT**GGCCAGAGCGGCC**AACGAGGACACGTGCTCCAC	*Sfi*I		
**SGUSF**	AT**GGCCATGTAGGCC**GCCAACTCCTACCGTACCTC	*Sfi*I	*uidA*	**gus^{S}-chs^{S}^chs^{A}-gus^{A}**
**SGUSR**[1]	AT**GGCCAGAGAGGCC**GTCGAGTTTACGCGTTGCTTCC	*Sfi*I		
**SCHSF**	AT**GGCCATGTAGGCC**ATTCGGAAACGTCACATGCATCT	*Sfi*I	At*CHS*	**chs^{S}-gus^{S}^gus^{A}-chs^{A}**
**SCHSR**[1]	AT**GGCCAGAGCGGCC**AACGAGGACACGTGCTCCAC	*Sfi*I		
**SgusF**	AT**GGCCTGAGTGGCC**ATTCGGAAACGTCACATGCATCT	*Sfi*I	*uidA*/At*CHS*	**chs^{A}-gus^{A}^gus^{S}-chs^{S}**
**SchsR**	AT**GGCCTTGTCGGCC**AACGAGGACACGTGCTCCAC	*Sfi*I		
**SchsF**	AT**GGCCTGAGTGGCC**GCCAACTCCTACCGTACCTCG	*Sfi*I	At*CHS*/*uidA*	**gus^{A}-chs^{A}^chs^{S}-gus^{S}**
**SgusR**	AT**GGCCTTGTCGGCC**GTCGATGTTACGCGTTGCTTCC	*Sfi*I		

[1] These primers were used twice to make different constructs.

Supplemental reference

Lee, Y., Kim, M., Han, J., Yeom, K.-H., Lee, S., Baek, S.H. and Kim, V.N. (2004) MicroRNA genes are transcribed by RNA polymerase II. *EMBO J.*, **23**, 4051-4060.

Supplemental Table S2. Changes in the abundance of rRNA-5S-derived reads after 5'PDE-mediated removal of uncapped RNA in Hi-Seq libraries. Changes reflect total number of corresponding reads in nine million reads per library. Sequence encoding rRNA-5S (Chr 2, 5782-5945) was used as a reference.

Hi-Seq Library	Number of reads	Change (%)
**RSeq 0**	4201	**35**
**ESeq 0**	2740	
**RSeq 1**	5935	**59**
**ESeq 1**	2409	
**RSeq 2**	3758	**48**
**ESeq 2**	1956	
**RSeq 3**	9938	**63**
**ESeq 3**	3632	
**RSeq 4**	4933	**50**
**ESeq 4**	2484	
**RSeq 5**	9223	**63**
**ESeq 5**	3389	
**RSeq 6**	8819	**49**
**ESeq 6**	4482	

Supplemental Table S3. (See separate Excel file.)
